# Brassicaceous roots as an unexpected diversity hot-spot of helotialean endophytes

**DOI:** 10.1186/s43008-020-00036-w

**Published:** 2020-08-11

**Authors:** Jose G. Maciá-Vicente, Meike Piepenbring, Ondřej Koukol

**Affiliations:** 1 Institute of Ecology, Evolution and Diversity, Goethe University Frankfurt, Max-von-Laue-Str. 13, 60438 Frankfurt am Main, Germany; 2 Integrative Fungal Research Cluster (IPF), Frankfurt am Main, Germany; 3 Department of Botany, Faculty of Science, Charles University, Benátská 2, 12801 Praha 2, Czech Republic

**Keywords:** 7 new species, Biogeography, *Cadophora*, Dark septate endophytes, *Ploettnerulaceae*, Symbiosis

## Abstract

A high number of fungal strains were isolated from roots of *Brassicaceae* species collected across western and southern Europe, resulting in an unexpectedly rich collection of *Cadophora* species. These isolates enable us to present a new and comprehensive view of the ecological, morphological, and phylogenetic traits of root-inhabiting members of this helotialean genus. We provide phylogenetic placement of all of our isolates based on a four-gene dataset, analyze their phenotypic traits in relation to their phylogenetic relationships, and infer the potential distribution ranges of the species by sequence comparisons with available databases. We consider seven well supported phylogenetic lineages as species new to science. Six further lineages probably also represent new species but remain undescribed due to the lack of diagnostic morphological characters. Our results show that *Cadophora*, as currently circumscribed, is paraphyletic and encompasses a broad spectrum of morphologies and lifestyles. Among the new species, only two (*C. ferruginea* and *C. constrictospora*) form phialides and conidia typical of *Cadophora*, three species (*C. echinata*, *C. gamsii* and *C. variabilis*) produce chains of swollen hyphal segments that may function as holoblastic conidia, and one species (*C. fascicularis*) produces chains of holoblastic ramoconidia and conidia. Ancestral state reconstruction analysis suggests that phialidic conidiogenesis evolved several times in *Cadophora s. lat.* from a putatively holoblastic common ancestor. Most *Cadophora* lineages are rare as estimated from the availability of sequence data, in spite of having relatively wide distribution ranges, whereas five lineages may represent endemic relationships given their restricted distributions. Our dataset, probably the most comprehensive available for *Cadophora*, nevertheless shows knowledge gaps concerning the phylogenetic relationships within this genus and highlights a need for further investigation.

## INTRODUCTION

Species of the genus *Cadophora* (*Ploettnerulaceae, Helotiales, Leotiomycetes*) are frequently associated with living plant roots and classified as so-called dark septate endophytes (DSE), a polyphyletic group of non-pathogenic, root-colonizing fungi characterized by melanized hyphae that are conspicuous when observing roots under the microscope (Jumpponen 2001; Sieber 2002; Addy et al. 2005). *Cadophora* has close phylogenetic affinities with other helotialean lineages with well-known root-associated habit, such as *Leptodontidium*, *Acephala*, and *Phialocephala* (with its teleomorph in *Mollisia*)*—*constituting the ‘mollisioid clade’ sensu Johnston et al. (2019)—and more distantly with mycorrhizal fungi in the *Hyaloscyphaceae* (Johnston et al. 2019). Whereas *Cadophora* species are also common as plant pathogens and colonizing substrata other than roots, such as wood or soil (Lagerberg et al. 1927; Bills 2004; Gramaje et al. 2011; Crous et al. 2017), several lines of evidence suggest a degree of adaptation in the genus toward a symbiotic association with plant roots or, leastwise, the possession of a genomic toolbox favoring the evolution of such a lifestyle. For example, representatives of the genus, or phylogenetically close strains, have been shown to have the ability to promote plant growth (Berthelot et al. 2016), to establish ericoid mycorrhizas (Bizabani and Dames 2015), or to engage in a mycorrhiza-like translocation of phosphorus to the host plant (Almario et al. 2017). Species related to *Cadophora* also possess genomes enriched in genes encoding carbohydrate active enzymes (CAZymes), proposed as likely markers of a plant-associated lifestyle (Hacquard et al. 2016; Almario et al. 2017; Knapp et al. 2018).

According to the original morphological generic concept, species of *Cadophora*, with the type species *C. fastigiata*, are characterized by solitary phialides with distinct hyaline, flaring collarettes (Lagerberg et al. 1927). However, deviations from this morphological standard exist, such as *C. orchidicola* with indehiscent conidia produced laterally on undifferentiated hyphae or terminally on unswollen or slightly swollen conidiogenous cells (Currah et al. 1987), or *C. antarctica* that produces chains of ramoconidia and conidia on holoblastic conidiogenous cells (Crous et al. 2017). The indistinct and largely variable morphological characters have led to significant confusion about the phylogenetic relationships and the boundaries of *Cadophora*. Soon after its creation, the genus was considered a synonym of *Phialophora* (*Chaetothyriales, Eurotiomycetes*) according to similar phialide morphology (Conant 1937), but it was later reinstated as a valid taxon within the *Helotiales* based on teleomorph links and molecular data (Gams 2000; Harrington and McNew 2003; Day et al. 2012). Other rearrangements of species, into or out of the genus, like those of *C. orchidicola* (formerly *Leptodontidium orchidicola*) or *Hyaloscypha finlandica* (formerly *C. finlandica*), relied on sequence data and amended morphology-based misplacements (Day et al. 2012; Fehrer et al. 2019). Sequence data have also led to the inclusion of *Cadophora* in the recently resurrected family *Ploettnerulaceae* (Jaklitsch et al. 2016; Johnston et al. 2019).

Presently, the genus *Cadophora* comprises 22 accepted species (www.indexfungorum.org) with diverse ecologies and modes of nutrition. The teleomorph stage is unknown for most *Cadophora* species, with the exception of several species producing mollisioid apothecia (Harrington and McNew 2003; Day et al. 2012; Pärtel et al. 2017; Ekanayaka et al. 2019). Further recent studies revealed new and hitherto overlooked species colonizing wood necroses (Bien and Damm 2020) and a high phenotypic diversity in the genus mirrored in new discoveries including the psychrotrophic *C. antarctica* isolated from Antarctic soil (Crous et al. 2017); *C. margaritata,* associated with the large poplar longhorn beetle (Linnakoski et al. 2018); and *C. helianthi* isolated from necrotic tissues in a wilting *Helianthus annuus* plant (Crous et al. 2019). Due to only subtle differences in morphology from other *Phialophora*-like fungi, molecular data are essential for species delimitation and identification in the genus.

Plants in the *Brassicaceae* are primarily non-mycorrhizal (Fitter 2005), but their roots harbor complex communities of endophytic fungi that include a great abundance and diversity of helotialean species, many of which are phylogenetically close to *Cadophora* (Almario et al. 2017; Glynou et al. 2018a, 2018b; Thiergart et al. 2019; Maciá-Vicente et al. 2020). Often, these *Cadophora*-like endophytes do not form evident parasitic interactions with the host, as appears to be common in endophytes from other lineages (Durán et al. 2017; Kia et al. 2017, 2018, 2019). Glynou et al. (2016) found a phylotype, identified as *Cadophora* sp. by ITS rDNA sequence data, to be widespread and abundant within roots of *Microthlaspi* spp. across western and southern Europe, as well as a number of other related phylotypes with a lower abundance and variable distribution breadths. In spite of their clustering in ITS-based phylotypes based on a 97% pairwise sequence similarity threshold, isolates in these groups show a high within-phylotype variation in their ITS sequences, their morphology, and their profiles of secondary metabolites production as compared to other lineages of fungal endophytes (Glynou et al. 2017; Maciá-Vicente et al. 2018). Altogether, this variability suggests that these helotialean fungi do not constitute a homogeneous population of widespread and generalist endophytes, but rather comprise a fragmentary array of lineages that have undergone adaptations to local conditions.

Here, we explore the phylogenetic and morphological diversity of 93 *Cadophora* isolates and related fungi isolated as root endophytes of *Microthlaspi* spp. (*Brassicaceae*), and provide descriptions for seven new species. In addition, we test for possible correlations of morphological species traits and ecological characters with phylogenetic relatedness. Lastly, we test the hypothesis that the species represent local (endemic) populations adapted to specific conditions.

## METHODS

### Fungal material

The fungi used in this study were isolated as root endophytes from *Microthlaspi* spp. plant individuals collected across western and southern Europe (Glynou et al. 2016). The only exception is isolate P6587, which was obtained from roots of *Arabidopsis thaliana* grown under laboratory conditions on a sterilized substrate containing a 1 g soil sample from a grassland in The Netherlands (Maciá-Vicente, unpubl.). The isolates were selected for their relationship to *Cadophora s. lat.*, based on comparisons of their ITS sequences with the UNITE database of reference fungal sequences (Kõljalg et al. 2005) as described in Glynou et al. (2016), and by BLAST searches against NCBI GenBank. UNITE Species Hypothesis (SH) codes (Kõljalg et al. 2013) were assigned to each isolate using ITS sequence similarities above 99.5%. Living cultures of all isolates have been deposited in the Integrative Fungal Research (IPF) culture collection hosted at Goethe University (isolate numbers starting with ‘P’ followed by a four-ciphers-number). A representative subset of the isolates has also been deposited in the CBS culture collection (Westerdijk Fungal Biodiversity Institute, Utrecht, The Netherlands). Dried cultures have been deposited in the Herbarium Senckenbergianum Frankfurt am Main (FR). The details of all isolates are provided in Table S1.

### Amplification and sequencing of phylogenetic markers

The ITS sequences for all isolates were available from previous studies (Glynou et al. 2016; Maciá-Vicente, unpublished). In addition, we used aliquots of genomic DNA stored at − 76 °C from all isolates to obtain partial sequences of the rDNA large (28S) subunit (LSU), the translation elongation factor 1-α (*tef1-α*), and the RNA polymerase II second largest subunit (*rpb2*). The LSU and *tef1-α* were amplified using primer pairs LR0R/LR7 (Hopple Jr and Vilgalys 1994) and EF1-728F/EF1-986R (Carbone and Kohn 1999), respectively, following procedures described by Maciá-Vicente et al. (2016). The *rpb2* was amplified using primers RPB2-6F/RPB2-7R (Ashrafi et al. 2018). Purified PCR products were bidirectionally sequenced using the PCR primers at the sequencing laboratory of the Biodiversity and Climate Research Centre (Frankfurt am Main, Germany), or at GATC Biotech (Konstanz, Germany). The assembled sequences have been deposited in GenBank, with accession numbers as listed in Table S1.

### Phylogenetic analyses

Sequences of reference strains from species related to our strains, including species types when available, were retrieved from NCBI GenBank and included in the datasets (Table S2). The sequence datasets for each locus (ITS, LSU, *tef1-α*, and *rpb2*) were first aligned using MAFFT v7.271 (Katoh and Standley 2013) using the G-INS-i parameters and manually edited and concatenated in the Geneious software package (Kearse et al. 2012). Genealogical concordance between the ITS, LSU, *tef1-α*, and *rpb2* datasets was assessed using R v3.6.1 (R Core Team 2019), by means of the partition homogeneity test (function *CADM.global*) available in package ape v5.3 (Paradis et al. 2004).

Because no significant support was found for incongruence among the tree topologies resulting from all four loci, a multilocus Maximum Likelihood (ML) phylogeny was obtained with RAxML v8.0 (Stamatakis 2014) using the general time reversible model of nucleotide substitution and the Γ model of rate heterogeneity (GTRGAMMA), allowing for different model parameter estimations per partition. The analysis was run on the CIPRES Science Gateway server (Miller et al. 2010) with nonparametric bootstrapping with 1000 replicates used for branch support. A complementary phylogeny based on Bayesian inference (BI) analysis was constructed with MrBayes v3.2.2 (Ronquist et al. 2012). The best-fit substitution model for each of the four loci was determined using the Bayesian information criterion in jModeltest v. 2.1.5 (Darriba et al. 2012). The selected models for the ITS, LSU, *tef1-α*, and *rpb2* were TIM2ef + I + G, TrN + I + G, HKY + G and TIM3ef + G, respectively. Two independent MCMC chains were run for 22 M generations with sampling every 100th generation, and a burn-in of 25% of the sampled trees was used. The average standard deviation of split frequencies estimating convergence reached the level of 0.007 at the end of the analysis. The alignments and trees are available at TreeBASE under accession S25942.

### Morphological characterization of isolates

The selected isolates were first recovered from long-term storage collection tubes containing cultures on corn meal agar (CMA, Sigma-Aldrich, St. Louis, MO, USA) covered with sterilized mineral oil (BDH Chemicals, Ltd., Poole, England), and plated on fresh CMA Petri plates. The isolates were further sub-cultivated on CMA, potato dextrose agar (PDA, AppliChem, Darmstadt, Germany), and 2% (w/v) malt extract agar (MEA, Carl Roth, Karlsruhe, Germany), by equidistantly placing on top of each medium three 5-mm-diam. plugs taken from two-week-old cultures on CMA. The plates with cultures were incubated upside-down at 25 °C in the dark. Three and seven days after inoculation, the diameter of all colonies (three per plate) were measured in two perpendicular directions, and the difference between the measurements at each day were used to calculate the rate of hyphal extension per day. Fifteen days after inoculation, the macro-morphological characteristics of the colonies on different media were recorded, including several measures of the colonies’ form, elevation, margin, and color (Table S1).

To estimate the quantity of conidia produced by each isolate, one-month-old cultures on CMA were used, with each Petri plate containing three equidistant colonies as described above. Three six-mm-diam. agar plugs, one per colony, were taken with a cork borer and placed in a 15-ml conical centrifuge tube with 1 ml 0.02% (v/v) Tween 20 (BDH Chemicals). The tubes were vortexed at maximum speed for 1 min and then the conidia in suspension were counted in a 10 μl aliquot using a haemocytometer. The number of conidia per colony surface area (conidiation) was calculated using the diameter of the agar plugs taken. Calibrated microscopical photographs of the conidia, when present, were captured with a Zeiss Axio Lab.A1 microscope equipped with an Axiocam Erc 5 s camera, and used to measure the length and width of around 20 conidia per isolate using the software Fiji/ImageJ (Schindelin et al. 2012). The dimensions of conidia were used to calculate conidial volume assuming ellipsoid shapes, according to formula 4 / 3 π length width^2^.

Isolates representing potentially distinct species based on the phylogenetic analysis were further used for a description of the micro-morphological characters in culture. Sporulating structures on the mycelium were mounted in Melzer’s reagent, lactic acid, or lacto-cotton blue and examined using differential interference contrast on an Olympus BX–51 (Olympus, Tokyo, Japan) with a digital camera Olympus DP72 (Olympus). Microscopic measurements are reported as the mean ± standard deviation of > 30 measurements.

### Ancestral state reconstruction of morphological and ecological characteristics

An ancestral state reconstruction of the target and reference strains was performed with the R package phytools v0.5 (Revell 2012), using a ML estimation with an equal-rates of transition model. Six discrete micro-morphological and ecological characteristics were considered (Tables S1 and S2): type of conidiogenesis (holoblastic, phialidic, unknown), type of conidia (branched, septate, non-septate, unknown), type of conidial attachment (chains, heads, single, unknown), substrate of origin (leaves, litter, roots, soil, stems, wood, unknown), aquatic lifestyle (yes or no), and life strategy (endophytic, parasitic, saprotrophic, saprotrophic/endophytic, unknown). For each character, the model returns a value of scaled likelihood (SL) per tree node. A subset of the Bayesian consensus tree containing only one specimen per species was used, in which the focal species that did not develop micro-morphological characteristics in culture were excluded. For reference species, data on the morphological and ecological characteristics were compiled from the literature references describing the selected strains (Table S2).

### Analysis of isolates’ distribution and morphological traits

Using only the focal isolates of this study, we evaluated whether any of the morphological traits measured were phylogenetically conserved by plotting their values for each lineage in a subset of the phylogenetic tree containing only one set of sequences per species. For quantitative traits (conidiation, conidial length-to-width ratio, growth rate, pigmentation, and conidial volume), the phylogenetic signal for the median values was calculated with the *K* statistic (Blomberg et al. 2003), using function *phylosig* of package phytools. The statistic measures conservation of traits among species, with *K* = 0 indicating absence of a phylogenetic signal, and *K* < 1 or *K* > 1 resemblance lower or higher than expected under Brownian motion evolution. Significance of *K* was assessed by comparing with a random shuffle of values at the tree tips.

We used a variation of principal component analysis (PCA) allowing for a mixture of qualitative and quantitative variables, available in R package PCAmixdata v3.1 (Chavent et al. 2014), to summarize the isolates’ morphological traits and assess their reliability to represent distinct species. Ancestral state reconstruction analysis was used to visualize the distribution of quantitative characters across the isolates’ phylogeny, as described above but using a Brownian motion model of character evolution. To investigate the relationship between the isolates’ phylogeny, their morphology, and their ecological origins, we first relied on Mantel correlograms (Legendre and Legendre 2012) built with function *mantel.correlog* of R package vegan v2.5–6 (Oksanen et al. 2015). Cophenetic distances among isolates were extracted from the phylogenetic tree and compared with Euclidean distances calculated from the coordinates of the morphology PCA (see above), or from the geographic coordinates of each location (geographic distance). To assess the relationships with soil physicochemical (soil distance) and climatic (climatic distance) conditions (Table S1), partial Mantel tests were applied to account for covariation with geographic distance.

The data and command line code to reproduce all these analyses is available at 10.6084/m9.figshare.12287816.

### Estimation of species’ distribution ranges

The distribution ranges of the species in the isolates’ selection were estimated by comparisons against publicly available geographical data on fungal isolations and sequence reads. The ITS sequences of all isolates were first compared using standalone BLAST (blastn version 2.2.31+) searches against a local copy of the NCBI nucleotide (nt) database, containing all fungal ITS sequences in GenBank as of August 2019. A second set of BLAST searches were performed against a selection of NCBI sequence read archive (SRA) objects, representing a selection of high-throughput ITS amplicon sequencing studies of fungi from soils or root samples worldwide, using the function blastn_vdb version 2.6.1+ of the NCBI SRA Toolkit (https://trace.ncbi.nlm.nih.gov/Traces/sra/sra.cgi?view=software). A summary of all databases used for these comparisons is provided in Table S3.

In both analyses, records from all BLAST matches with a minimum of 98% identity, and with a minimum alignment length of 200 nt in the case of SRA objects, were retained. The geographical locations of the records, as latitude/longitude coordinates or countries, were obtained from the sequences’ metadata, when available, using custom python scripts. When only data on country but no coordinates were available, the geographical coordinates for the country’s centroid were obtained using the R package maps version 3.3.0 (Becker et al. 2018). The geographical distribution of records was visualized using functions in the R package ggplot2 version 2.3.2.0 (Wickham et al. 2019) and maps of package rnaturalearth version 0.1.0 (South 2017). The command line code for these analyses is available at https://doi.org/10.6084/m9.figshare.12287816.

## RESULTS

### Molecular phylogenetic analyses

Ninety-three isolates originating as root endophytes from brassicaceous hosts were included in this study. Affinities with members of the helotialean genus *Cadophora* were recognized based on BLAST comparisons of ITS sequences against the UNITE database (Table S1). Sequences from a selection of 75 strains with reliable species-level identifications were retrieved from NCBI GenBank and used as references for phylogenetic comparison (Table S2). The alignment of partial sequences of ITS, LSU, *rpb2*, and *tef1-α* had 2767 characters (638, 885, 725, and 519, respectively).

**Fig. 1:**
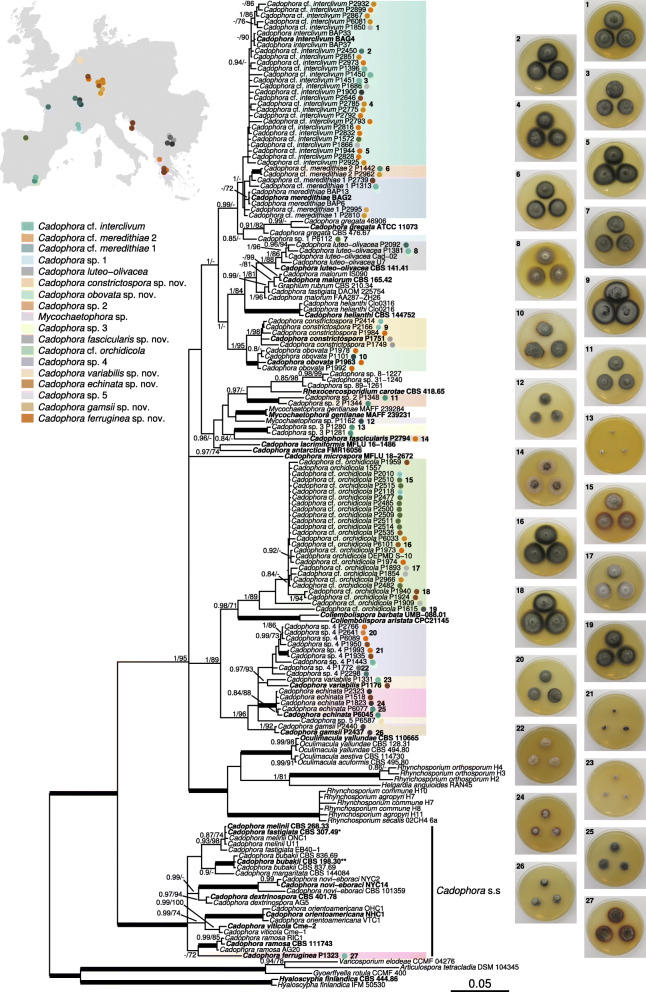
Phylogenetic relationships of the isolates of this study and reference species related to the genus *Cadophora*. The tree shows a topology based on Bayesian inference analysis of concatenated ITS, LSU, *tef1-α*, and *rpb2* partial sequences. Node support values correspond to posterior probabilities (PP) obtained by Bayesian inference, and to bootstrap (BS) analyses based on 1000 replicates of a maximum likelihood phylogeny (PP/BS). Only PP and BS values above 0.8 and 70 are shown, respectively, and thickened branches indicate strong support with PP = 1 and BS > 99. The isolates of this study are indicated by colored bullets next to the isolates names, with colors representing their geographical origin (see color key in map inset). The species these isolates belong to are highlighted by colored clades (see color key with species names). Strain names highlighted in bold face correspond to species types, or isolates putatively representing them. The *Cadophorasensu stricto* (s.s.) clade is demarcated with a bracket. Examples of some of the isolates’ colony aspect are shown in the pictures rightwards to the tree, depicting 15-day-old pure cultures of individual isolates (see number referring to isolates) on 2% malt extract agar. *, *C. fastigiata* CBS 307.49, although not a type specimen, is considered to represent well the genus type by multiple authors (see Discussion). **, *C. bubakii* CBS 198.30 is an isotype

Both the ML and BI phylogenetic trees agreed in showing a clear paraphyly in the genus *Cadophora*, with representative species scattered across four main clades (Fig. 1), e.g. those including *C. interclivum*, *C. meredithiae*, and *C. gregata*; *C. luteo-olivacea*, *C. malorum*, and *C. helianthi*; *C. orchidicola*; and the type species of the genus, *C. fastigiata*, alongside *C. novi-eboraci*, *C. orientoamericana*, and *C. ramosa*; which were interspersed with species of the genera *Collembolispora*, *Mycochaetophora*, *Oculimacula*, *Rhexocercosporidium*, and *Rhynchosporium*. Branch support was strong in some of these high-level clades based on both ML bootstrap (BS) and Bayesian posterior probability (PP), although in other cases (e.g., for the cluster containing *C. interclivum* and *C. meredithiae*) only PP was high (Fig. 1). The clade containing the type species *C. fastigiata* appears to be basal to all other clades with *Cadophora* species, constituting a well-defined clade that henceforth we refer to as *Cadophora s. str.* Based on the position of the ex-type of *Mollisia dextrinospora* within *Cadophora s. str.*, we propose its combination into *Cadophora*.

The focal isolates of this study were grouped into 18 clades with variable branch support (Fig. 1). The multilocus phylogeny showed a better resolution of relationships among isolates than clustering into operational taxonomic units (OTUs) based on 97–98% ITS sequence similarity, common in diversity barcoding studies, because isolates within each OTU were scattered across distant clades of the tree (Fig. S1). OTUs based on 99% ITS similarity, however, matched relatively well the clades defined by the multilocus analysis (Fig. S1). Most isolates grouped in two clades, either together with multiple strains of *C. interclivum* and its sister species *C. meredithiae* (including the ex-type strains) or with two strains (DEPMD S-10 and 1557) tentatively identified as *C. orchidicola*. The *C. interclivum*/*C. meredithiae* clade comprised multiple lineages with medium or no support, including a strongly supported sub-clade formed by isolates P1442 and P2962 that is treated separately in further analyses. In view of phenotypic differences of our isolates with both *C. interclivum* and *C. meredithiae*, we tentatively named these species *C.* cf. *interclivum* and *C.* cf. *meredithiae*. The isolates in the *C. orchidicola* clade, were in turn identified as *C.* cf. *orchidicola*, given the absence of sequence data for the type specimen of this species. These species-level clades were strongly supported by PP, whereas support was low or only moderately high (89% in *C.* cf. *orchidicola*) based on BS. Other two isolates, P1381 and P2092, were classified as *C. luteo-olivacea* because of their grouping in a lineage including the ex-type sequence of this species (Fig. 1).

The other 13 clades grouped separately from other known species (Fig. 1), and here we propose seven of them as new species (Fig. 1). The remaining six lineages (*Cadophora* sp. 1, *Cadophora* sp. 2*, Cadophora* sp. 3, *Cadophora* sp. 5, and *Mycochaetophora* sp.) apparently represent new species but are not described as new here due to the lack of discriminating morphological characters of many cultures which remained sterile even after prolonged cultivation on diverse media.

### Ancestral state reconstruction of morphological and ecological characteristics

**Fig. 2:**
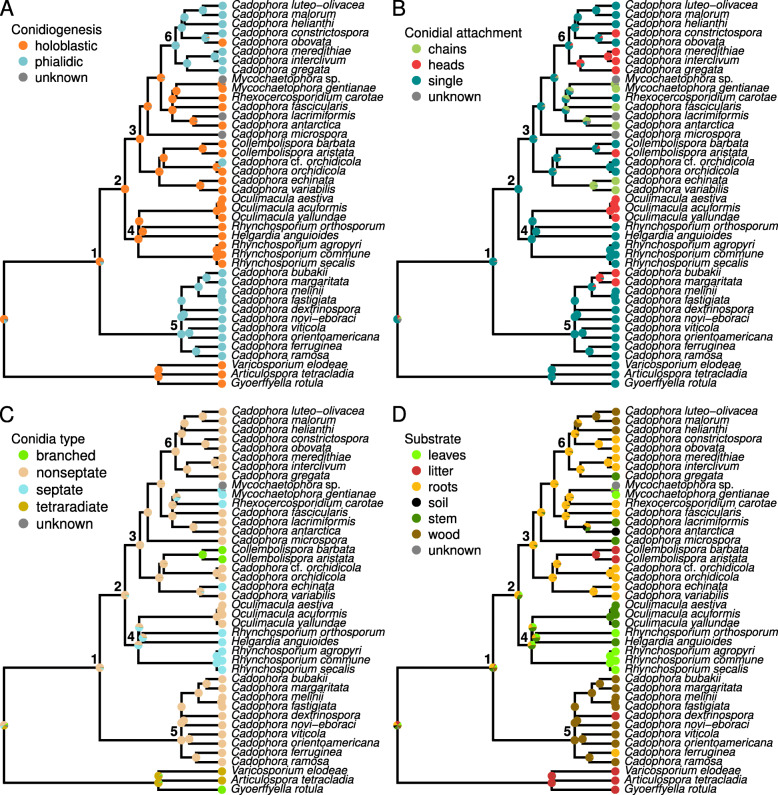
Ancestral character state reconstruction in *Cadophora* and allied species. The underlying tree is a subset of the Bayesian consensus tree shown in Fig. 1, containing only one specimen per species, and not including the focal species that did not develop micro-morphological characteristics in culture. **a** type of conidiogenesis. **b** type of conidia. **c** type of conidial attachment. **d** substrate of origin. Proportional likelihoods for each character state are represented by pie charts at the tree nodes. Tree nodes marked with numbers are described in the main text

The reconstruction of the evolution of three morphological and three ecological characters reflected the paraphyly in *Cadophora* described above (Fig. 2; Fig. S2). Morphologically, the cluster hosting the genus type *C. fastigiata* (node 5 in Fig. 2) forms a stable group with species that have phialidic conidiogenesis and that produce single non-septate conidia, with the only exception of *C. bubakii* and *C. margaritata* that produce conidia in heads (Fig. 2a–c). Contrarily, the ancestor of all other species (node 2 in Fig. 2) likely had a holoblastic conidiogenesis (SL = 0.992) that was conserved in descendant lineages, with a secondary evolution of phialidic conidiogenesis in the ancestor (SL = 0.978) of the clade containing *C. malorum*, *C. luteo-olivacea*, *C. interclivum*, and *C. gregata* (node 6 in Fig. 2a). Whereas the conidial attachment and the type of conidia in this cluster appear to be highly variable across lineages, all species in this cluster may have evolved from an ancestor (node 2 in Fig. 2) with single (SL = 0.974) non-septate conidia (SL = 0.899).

Ecologically, all *Cadophora* and allied genera appear to have evolved from a fungus with a terrestrial (SL = 0.991) and most likely parasitic habit (SL = 0.883, node 1 in Fig. 2), with a subsequent adaptation to aquatic environments only in *Collembolispora* (also showing branched conidia characteristic of waterborne fungi), and the evolution of saprotrophic and/or endophytic lifestyles in multiple instances (Fig. S2). Whereas it is difficult to establish the substrate preferences of the common ancestor for all *Cadophora* and allied genera (node 1 in Fig. 2), different lineages apparently radiated to exploit different substrata, mostly plant organs (Fig. 2d), which agrees with the documented plant pathological lifestyle of some *Cadophora* species. Species of *Cadophora s. str.* (node 5 in Fig. 2) show a preference towards wood colonization and evolved from a common ancestor that most probably lived on wood (SL = 0.999); whereas the remaining *Cadophora* species (node 3 in Fig. 2) may have evolved from a mostly root-colonizing fungus (SL = 0.883), but displaying either adaptations to other substrata, or a flexible lifestyle favoring the colonization of multiple niches (Fig. 2d).

### Morphological characters of isolates in culture

**Fig. 3:**
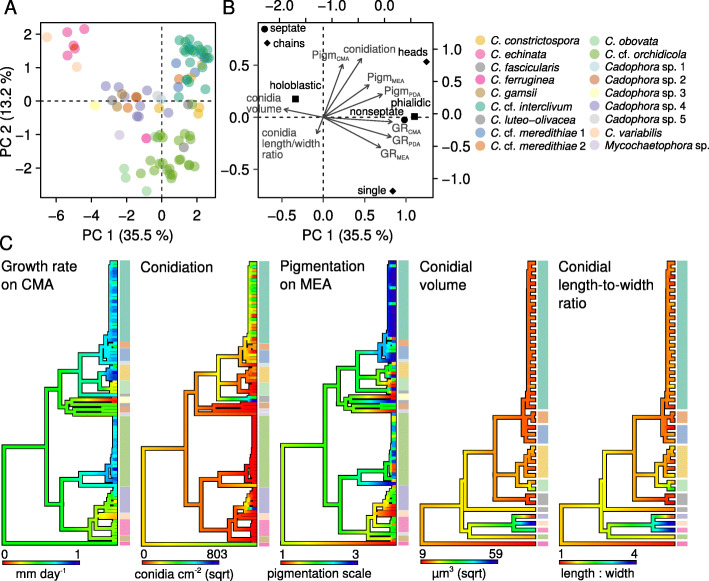
Morphological characterization of the isolates of this study. **a** Principal component analysis (PCA) ordination of isolates according to their quantitative morphological characters. Each point represents one isolate, with color indicating the species to which it belongs to. **b** PCA scores showing the contribution of each morphological character to the separation of the isolates, as indicated by the direction and magnitude of the arrows. **c** Distribution of selected quantitative characters across the isolates’ phylogeny. Colors next to tree tips indicate the isolates’ species (see color key). Note that trees for conidial volume and conidial length-to-width ratio contain only a subset of the species that produced conidia in culture. For trees showing all the characters included in **b**, see Fig. S3. Abbreviations: GR, growth rate on each agar medium (indicated by subscript); Pigm, degree of dematiaceous pigmentation on each agar medium (indicated by subscript)

Ordination of isolates according to both quantitative and categorical morphological traits in culture explained a mere 20% of overall variation, with no clear separation among clades (Fig. S3). This was largely due to the inclusion of categorical variables in the analysis that are not informative for species discrimination. The removal of most categorical characters from the ordination, except for type of conidiogenesis, type of conidia, and type of conidial attachment that appeared to carry phylogenetic signal, increased the proportion of variation explained to 47.7%, discriminating among several of the species (Fig. 3a). Separation among species was strongest based on growth rates in all media and the presence of septa in conidia, and to a lesser extent by the degree of pigmentation on PDA and MEA and conidial volume, explaining differences mainly along the PC 1 axis (Fig. 3b). These drove differences between species like *C.* cf. *interclivum* and *C.* cf. *orchidicola*, and slow-growing ones such as *Cadophora* sp. 3 or *C. echinata* (Fig. 1a, c; Fig. S4). The type of conidiogenesis, conidial attachment, pigmentation on CMA medium, production of conidia on agar medium, and the conidial length-to-width ratio explained most variation along the PC 2 axis (Fig. 3b), mainly determining differences between the fast-growing species *C.* cf. *interclivum* and *C.* cf. *orchidicola* (Fig. 3a, c; Fig. S4). In spite of the marked differences in quantitative characters between particular species, none of them appeared to be conserved across the phylogeny of the group (Table S4), indicating a lack of strong selective pressure on these traits. However, visualization of the distribution of characters across the isolates’ phylogeny showed marked differences between particular clades (Fig. 3c), following the patterns described by the PCA ordination (e.g. fast growth-rates for *C.* cf. *interclivum*/*meredithiae*, *C. constrictospora*, and *C.* cf. *orchidicola*, as compared to others; consistently dark pigmentation in *C.* cf. *interclivum*; or high conidial length-to-width ratio in *C. fascicularis*). The spores could not be measured for several species that did not produce conidia in culture (see conidiation in Fig. 3c).

### Geographic and ecological origins of isolates

The isolates originated from 30 sampling sites in seven European countries, separated a minimum of 2 km from one another (Fig. 1). Most clades including the focal isolates comprised isolates originating from geographically distant locations, separated by hundreds to thousands of km, indicating a low level of endemism in the species. Locally restricted distributions were only found in clades represented by individual isolates or by couples, potentially clonal isolates, likely reflecting a low representation in our collection of isolates rather than true geographic endemism. Phylogenetic relatedness between isolates had a low correlation with geographical distance also according to a Mantel test (*r* = 0.07, *P* = 0.01), indicating a lack of spatial structure in these fungal populations. Likewise, partial Mantel tests accounting for spatial distance showed that similarity in climatic factors or in physico-chemical characteristics of soils of isolation sites were poorly associated with phylogenetic similarity among isolates (*r* = 0.08, *P* = 0.047 and *r* = 0.05, *P* = 0.089, respectively).

### Species distribution ranges

**Fig. 4:**
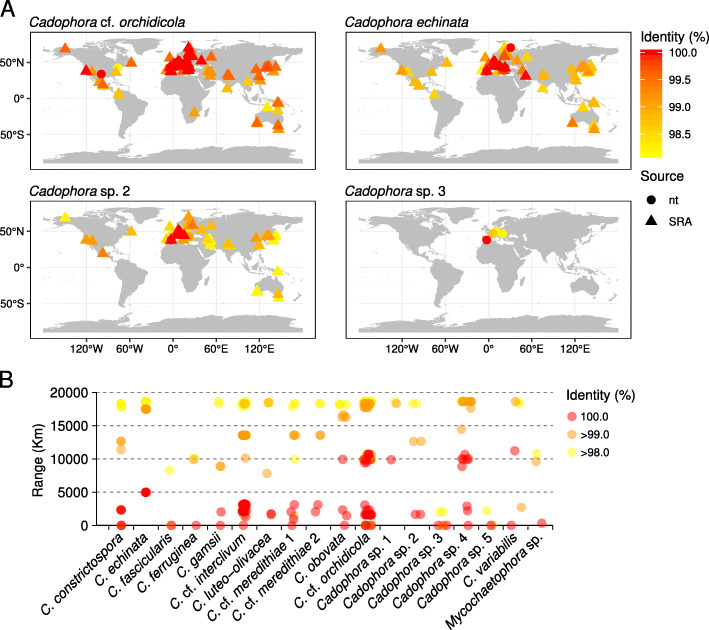
Potential worldwide distribution of the *Cadophora* species target of this study as inferred by BLAST comparisons against the NCBI GenBank’s nucleotide database (nt), and selected high-throughput ITS amplicon sequencing datasets available at the sequence reads archive (SRA; Table S3). **a** Maps showing the worldwide distribution of BLAST matches for selected *Cadophora* species (see Fig. S4 for maps with all the species in this study). Each map shows the BLAST search results for all the isolates within each species. Points represent the geographic locations of BLAST matches, either from the nt or the SRA database (indicated by point shape). Only BLAST matches with percent identity above 97% are shown, with points color indicating percent identity value. **b** Species distribution ranges based on BLAST results at 98, 99, or 100% percent identity. Ranges correspond to the farthest distance between two BLAST matches in the map, at each percent identity. For each species, each point corresponds to the range calculated for each isolate within the species

Comparison of ITS sequences from all isolates with GenBank records and sequences from high-throughput amplicon sequencing studies of fungal diversity worldwide (Table S3) showed potential distribution ranges largely restricted to the northern hemisphere (Fig. 4, Fig. S5). We evaluated BLAST results with a minimum of 97% sequence similarity to account for undefined ITS-based species delimitations, but only values above 99% are likely to approximate actual species boundaries (Fig. S1), with 100% values providing conservative assignments. At 100% similarity, most species showed a restricted European distribution, with matches both in the nt and SRA databases spanning a maximum of 5000 km (Fig. 4b, Fig. S5). Only *C. obovata*, *C.* cf. *orchidicola*, *Cadophora* sp. 1, *Cadophora* sp. 4, and *C. variabilis* surpassed that threshold, with reports in either North America and/or Asia, but never south from the equator (Fig. 4b, Fig. S5). In contrast to these species, *C. fascicularis*, *C. ferruginea*, *Cadophora* sp. 3, *Cadophora* sp. 5, and *Mycochaetophora* sp. showed very restricted ranges with respect to GenBank records, often even at low percent identities (Fig. 3b, Fig. S4).

## TAXONOMY

**Cadophora dextrinospora** (Korf) Koukol & Maciá-Vicente, **comb. nov.**

MycoBank: MB 834822.

*Basionym*: *Mollisia dextrinospora* Korf, *Mycotaxon***10**: 462 (1980).

*Note:* The affinity of *M. dextrinospora*, distinct by dextrinoid reaction of its ascospores, to the genus *Cadophora*, and notably to *C. fastigiata*, was recognized already by Greenleaf and Korf (1980) and later confirmed by Gams (2000). The combination into *Cadophora* is further supported by the current placement of *Mollisia* in a lineage distinct from *Ploettnerulaceae* (Jaklitsch et al. 2016), and its connection with *Phialocephala* (Tanney et al. 2016).

**Fig. 5:**
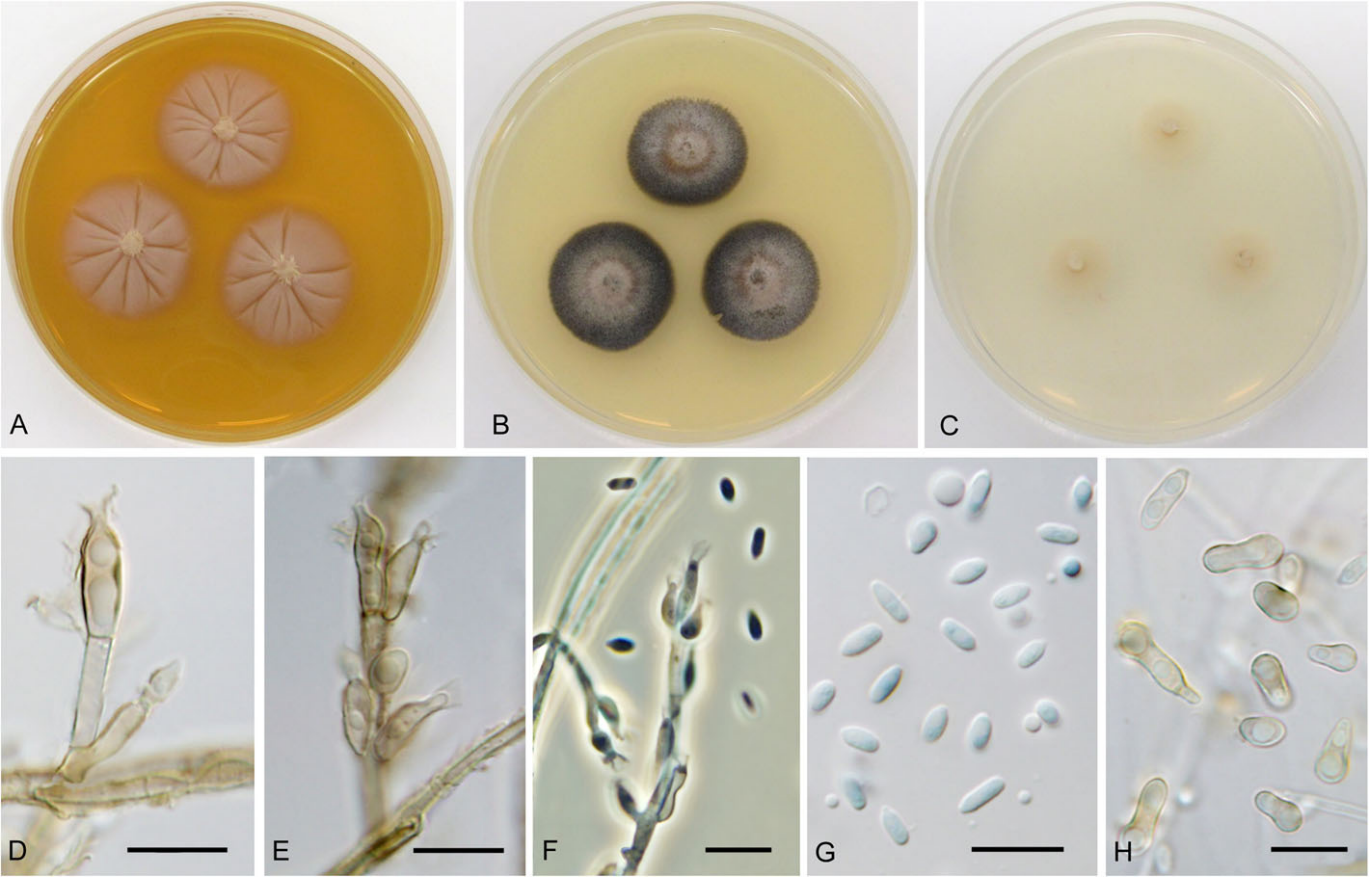
*Cadophora constrictospora* (CBS 146371 – ex-type culture). **a**–**c** 15-day-old colonies on MEA, PDA, and CMA, respectively. **d–e** conidiophore with terminal and intercalary phialides. **f** conidiophore with phialides and conidia in phase contrast. **g** primary conidia. **h** secondary conidia. Bars: 10 μm

**Cadophora constrictospora** Koukol & Maciá-Vicente, **sp. nov.** – Figs. 1, 5.

MycoBank: MB 834823.

*Etymology*: After the production of secondary conidia that are distinctly constricted.

*Diagnosis*: *Cadophora constrictospora* differs from *C. fastigiata* in having distinctly constricted secondary conidia.

*Type*: **Bulgaria**: Nepraznentsi, 42.67 N 022.84 E, 740 m a.s.l., endophytic in roots of *Microthlaspi* sp., 14 May 2013, *T. Ali & S. Ploch* [isol. K. Glynou] (FR 0255157 – holotype; P1751 = CBS 146371 – ex-type cultures: GenBank accessions: ITS = KT269023, LSU = MN339369, *tef1-α* = MN325874, *rpb2* = MN367280).

*Description*: *In culture* – *Colonies* on MEA reaching 28–30 mm diam after 10 d, on PDA and CMA reaching 27–28 mm and 17–18 mm diam, respectively. *Mycelium* septate, hyphae subhyaline to pale brown, forming fascicles up to 18 μm diam, hyphae 2–4 μm thick. *Conidiophores* pale brown, straight, unbranched, producing terminal or intercalary fascicles of phialides, rarely absent. *Conidiogenous cells* phialidic, pale brown, smooth, lanceolate, with distinct collarette up to 3.5 μm long, (8.5–)10–14.5(− 17.5) × 2–4 μm wide. *Conidia* of two types: primary conidia hyaline, ellipsoid to narrowly ellipsoid, smooth, nonseptate, with distinct projection on the basis, forming compact heads on the top of phialides, 4–6.5 μm long and 2–2.5 μm wide, the mean conidium length/width ratio 2.3:1; secondary conidia pyriform, spathulate to ossiform secondary conidia with distinct constrictions in the middle are formed in older cultures, 6.5–10(− 12.5) × 3–5 μm wide, the mean conidium length/width ratio 2:1.

*Note:* The newly described species is morphologically most similar to *C. fastigiata* that has phialides and conidia of similar shape and size, however *C. constrictospora* produces also large secondary conidia with distinct constriction in cultures more than 30 d old. These secondary conidia do not seem to have distinct origin, so that we assume that they are produced by ageing phialides. The morphological similarity is however result of convergence, since the two species are largely unrelated (Fig. 1).

**Fig. 6:**
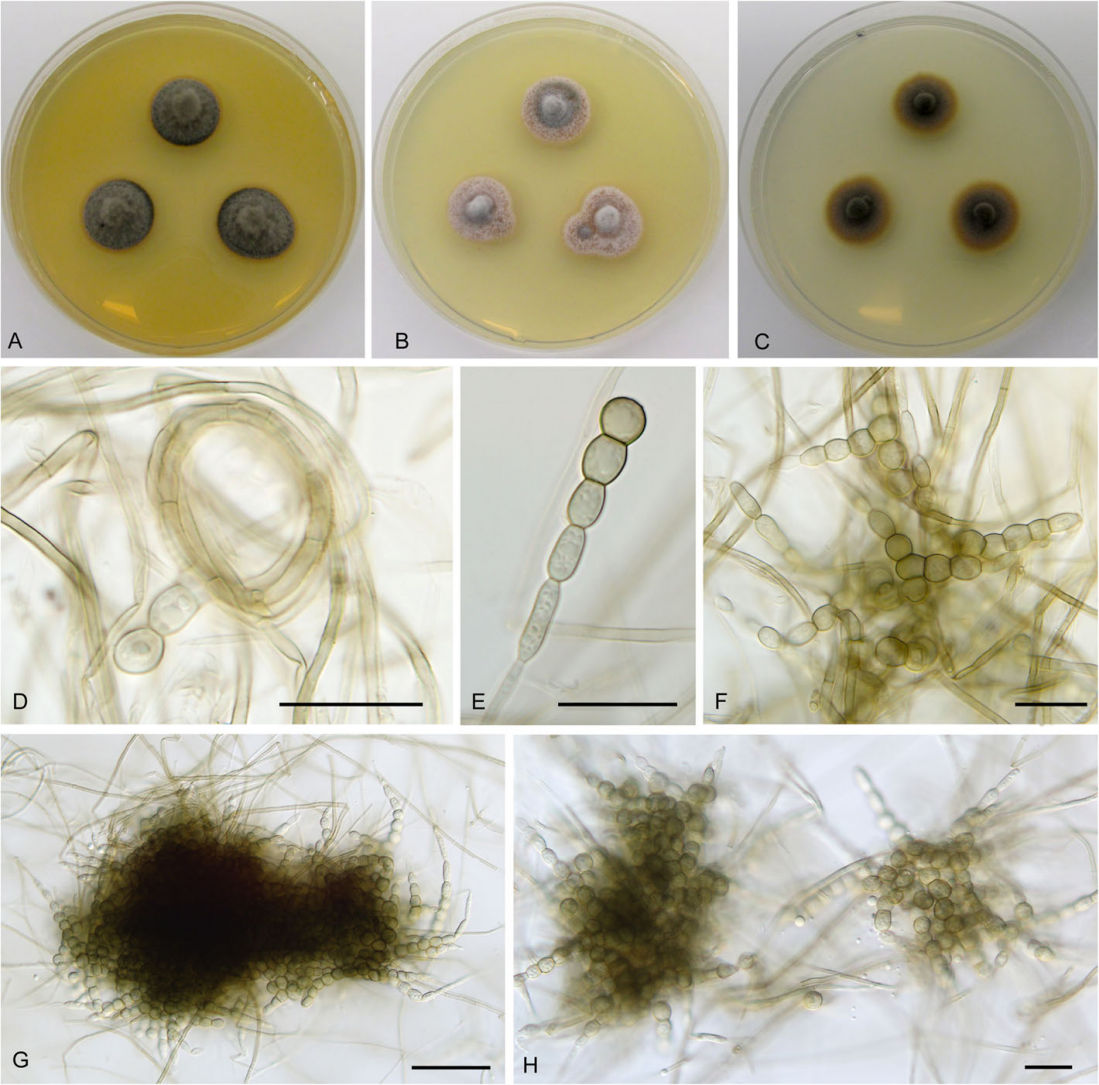
*Cadophora echinata* (CBS 146383 – ex-type culture). **a**–**c**, 15-day-old colonies on MEA, PDA, and CMA, respectively. **d** young conidium formed on hyphal coil. **e** terminally formed conidium. **f–h** complex conidia at various stages of maturity. Bars: D – E = 10 μm, F – H = 20 μm

**Cadophora echinata** Koukol & Maciá-Vicente, **sp. nov***.* – Figs. 1, 6.

MycoBank: MB 834828.

*Etymology*: After the frequent production of conidia having echinate appearance.

*Diagnosis*: *Cadophora echinata* is distinct in producing complex holoblastic conidia that branch into compact echinate bodies up to 150 μm large.

*Type*: **Spain**: Puebla de Don Fadrique, 38.05 N 002.54 W, 1612 m a.s.l., endophytic in roots of *M. perfoliatum*, 2 May 2013, *J.G. Maciá-Vicente* (FR 0255199 – holotype; P6045 = CBS 146383 – ex-type cultures; GenBank accessions: ITS = KT270239, LSU = MN339428, *tef1-α* = MN325932, *rpb2* = MN367267).

*Description*: *In culture* – *Colonies* on MEA reaching 15 mm diam after 10 d, on PDA and CMA reaching 14–16 mm and 15–16 mm diam, respectively. *Mycelium* septate, hyphae hyaline to dark brown, rarely forming coils up to 38 μm diam, hyphae 2–3 μm wide. *Conidiophores* not developed. *Conidiogenous cells* integrated, terminal, holoblastic, monoblastic, smooth, cylindrical, 3–5 μm wide. *Conidia* holoblastic complex, originating as chains of fusiform, oval to almost globose cells strongly constricted at the septa, pale brown and becoming darker towards the centre, later branching repeatedly and forming compact tuft-like bodies up to 150 μm diam, individual cells 5.5–9 × 5–9 μm wide.

*Additional material studied*: **Croatia**: Gospić, 44.46 N 015.40 E, 755 m a.s.l., endophytic in roots of *M. erraticum*, 4 May 2013, *T. Ali* [isol. K. Glynou] (FR 0255154; P1518 – culture). **– Bulgaria**: Divlya, 42.57 N 022.69 E, 685 m a.s.l., endophytic in roots of *M. erraticum*, 14 May 2013, *T. Ali & S. Ploch* [isol. K. Glynou] (FR 0255159; P1823 – culture); ibidem (FR 0255182; P2323 – culture). **– Spain**: Puebla de Don Fadrique, 38.05 N 002.54 W, 1612 m a.s.l., endophytic in roots of *M. perfoliatum*, 2 May 2013, *J.G. Maciá-Vicente* (FR 0255200; P6077 – culture).

*Note:* For comments on *C. echinata* see the note under *C. gamsii*.

**Fig. 7:**
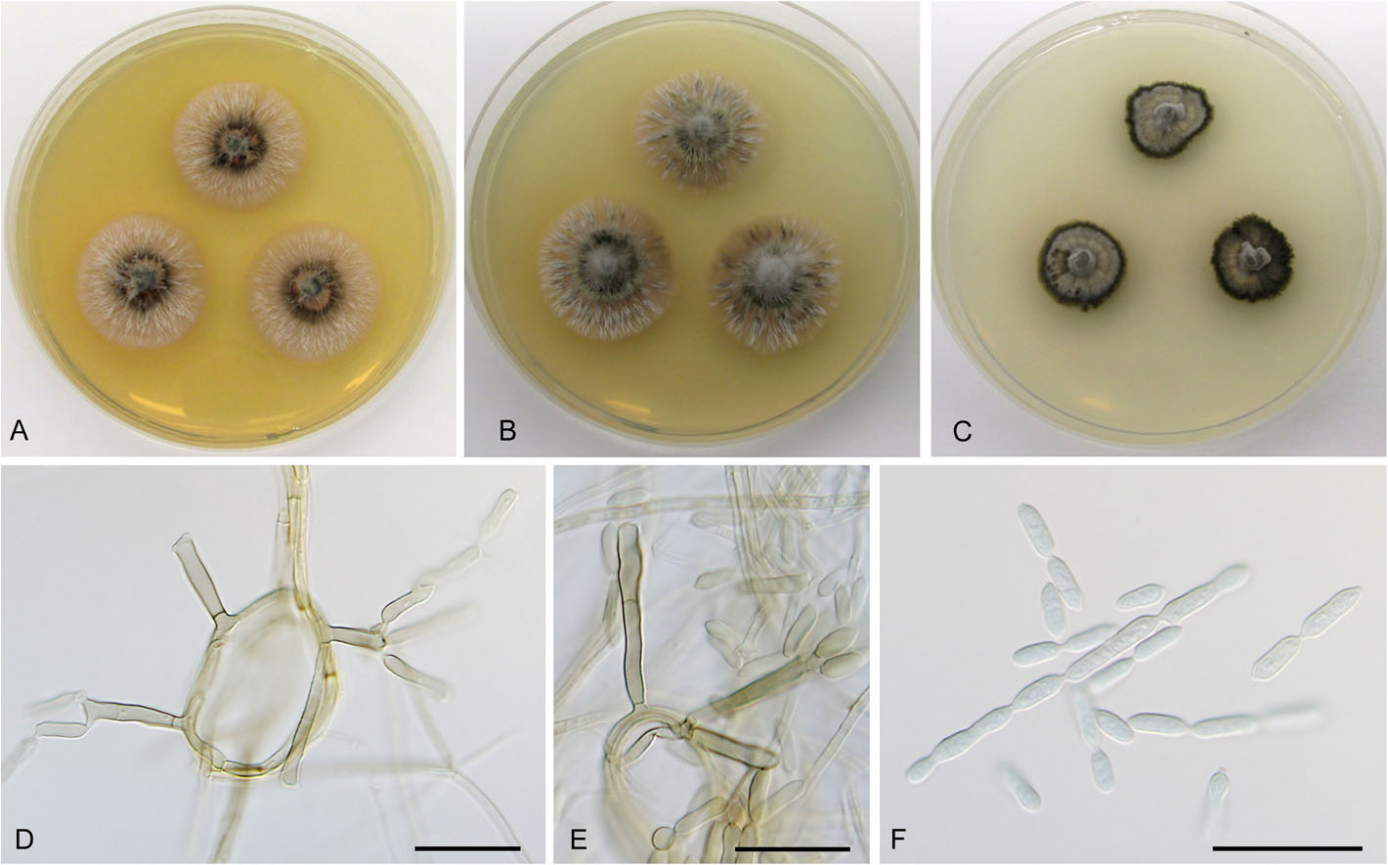
*Cadophora fascicularis* (CBS 146382 – ex-type culture). **a** – **c** 15-day-old colonies on MEA, PDA, and CMA, respectively. **d** – **e** conidiophores formed on hyphal coils. **f** chains of conidia and ramoconidia. Bars: 20 μm

**Cadophora fascicularis** Koukol & Maciá-Vicente, **sp. nov**. – Figs. 1, 7.

MycoBank: MB 834824.

*Etymology*: After the production of ramoconidia in apical fascicles.

*Diagnosis*: Morphologically similar to *C. antarctica*, but with conidiophores with holoblastic cylindrical conidiogenous cell producing chains of ramoconidia and conidia.

*Type*. **Germany**: Darstadt, 49.68 N, 010.00 E, 278 m a.s.l., endophytic in roots of *M. erraticum*, 6 June 2013, *K. Glynou & J.G. Maciá-Vicente* [isol. K. Glynou] (FR 0255219 – holotype; P2794 = CBS 146382 – ex-type cultures; GenBank accessions: ITS = KT269992, LSU = MN339414, *tef1-α* = MN325918).

*Description*: *In culture* – *Colonies* on MEA reaching 17–18 mm after 10 d, on PDA and CMA reaching 16–17 mm and 14–15 mm, respectively. *Mycelium* septate, hyphae hyaline to dark brown, frequently forming coils up to 50 μm in diam, hyphae 2–2.5 μm in diam. *Conidiophores* growing from vegetative hyphae or hyphal coils, straight, smooth, consisting of 1–2 cells terminated by a conidiogenous cell, up to 50 μm long and 3–4 μm wide, frequently reduced to the conidiogenous cell. *Conidiogenous cells* holoblastic, polyblastic, smooth, cylindrical, cuneiform to almost club shaped with several loci at the apex, (11)12.5–18(20.5) μm long and up to 3–5 μm wide in the upper part. *Ramoconidia* holoblastic 0(− 1) septate, pale brown to hyaline, sometimes paler towards the apex, usually forming fascicles on the top of the conidiogenous cell, smooth, straight to slightly curved, subcylindrical to fusiform, not constricted at the septum, (6.5)10.5–16(20) μm long and 2.5–4 μm wide. *Conidia* hyaline, smooth, elongated lemon shaped to fusiform with slight constriction in the middle, forming short chains, (6.5)8–11(15) μm long and 2–3 μm wide, the mean conidium length/width ratio 3.5:1.

*Note: Cadophora fascicularis* differs from most *Cadophora* species by a lack of phialidic conidiogenous cells. It is morphologically somewhat comparable to *C. antarctica* that also produces chains of ramoconidia and conidia on holoblastic conidiogenous cells. However, *C. fascicularis* has well developed conidiophores, ramoconidia, and conidia are more elongated than in *C. antarctica.*

**Fig. 8:**
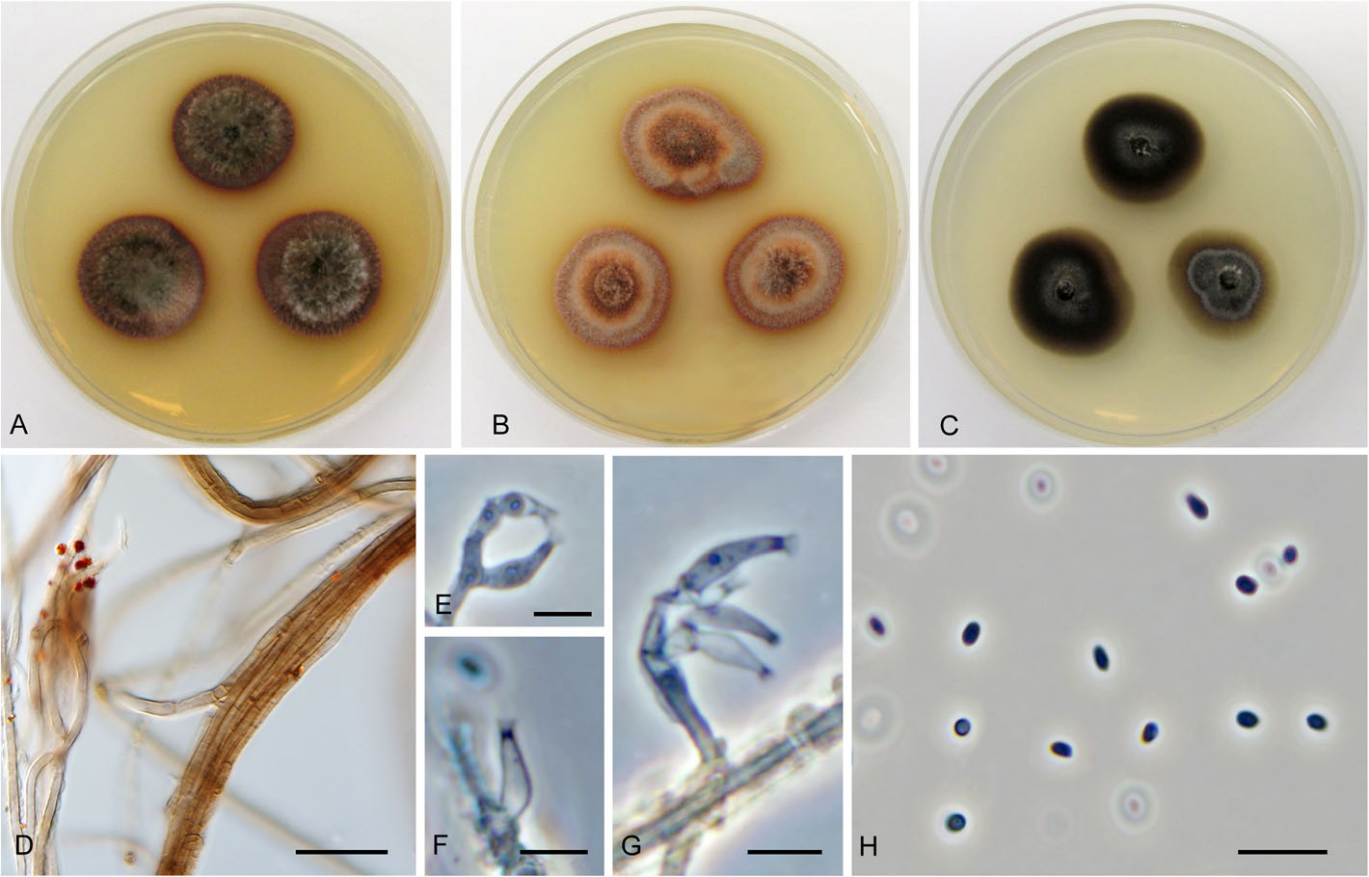
*Cadophora ferruginea* (CBS 146363 – ex-type culture). **a** – **c** 15-day-old colonies on MEA, PDA, and CMA, respectively. **d** hyphal fascicles. **e** – **g**, phialides in phase contrast. **h**, conidia in phase contrast. Bars: D = 20 μm, E – G = 5 μm, H = 10 μm

**Cadophora ferruginea** Koukol & Maciá-Vicente, **sp. nov.** – Figs. 1, 8.

MycoBank: MB 834826.

*Etymology*: After the production of reddish globules and mycelium.

*Diagnosis*: Morphologically distinct from *C. fastigiata* in having smaller phialides with indistinct collarette and reddish mycelium in culture.

*Type:***Spain**: Puebla de Don Fadrique, 38.04 N 002.48 W, 1630 m a.s.l., endophytic in roots of *M. perfoliatum*, 2 May 2013, *J.G. Maciá-Vicente* (FR 0255144 – holotype; P1323 = CBS 146363 – ex-type cultures; GenBank accessions: ITS = KT268618, LSU = MN339356, *tef1-α =* MN325861).

*Description*: *In culture* – *Colonies* on MEA reaching 18–20 mm diam after 10 d, on PDA and CMA reaching 18–19 mm and 16–22 mm diam, respectively. *Mycelium* septate, hyphae subhyaline to reddish, forming fascicles up to 15 μm diam, hyphae 2–4 μm wide. *Conidiophores* hyaline, straight, branched and producing terminal fascicle of phialides, rarely absent. *Conidiogenous cells* phialidic, hyaline, smooth, with indistinct collarette (that may disappear at older phialides), 6–10 × 2–3 μm wide. *Conidia* hyaline, ellipsoid, smooth, nonseptate, forming compact heads on the top of phialides 3–5 × 1.5–2.5 μm wide, the mean conidium length/width ratio 1.8:1.

*Note: Cadophora ferruginea* belongs among other phialidic members of this genus, but produces phialides that are generally smaller and may be distinguished by reddish mycelium.

**Fig. 9:**
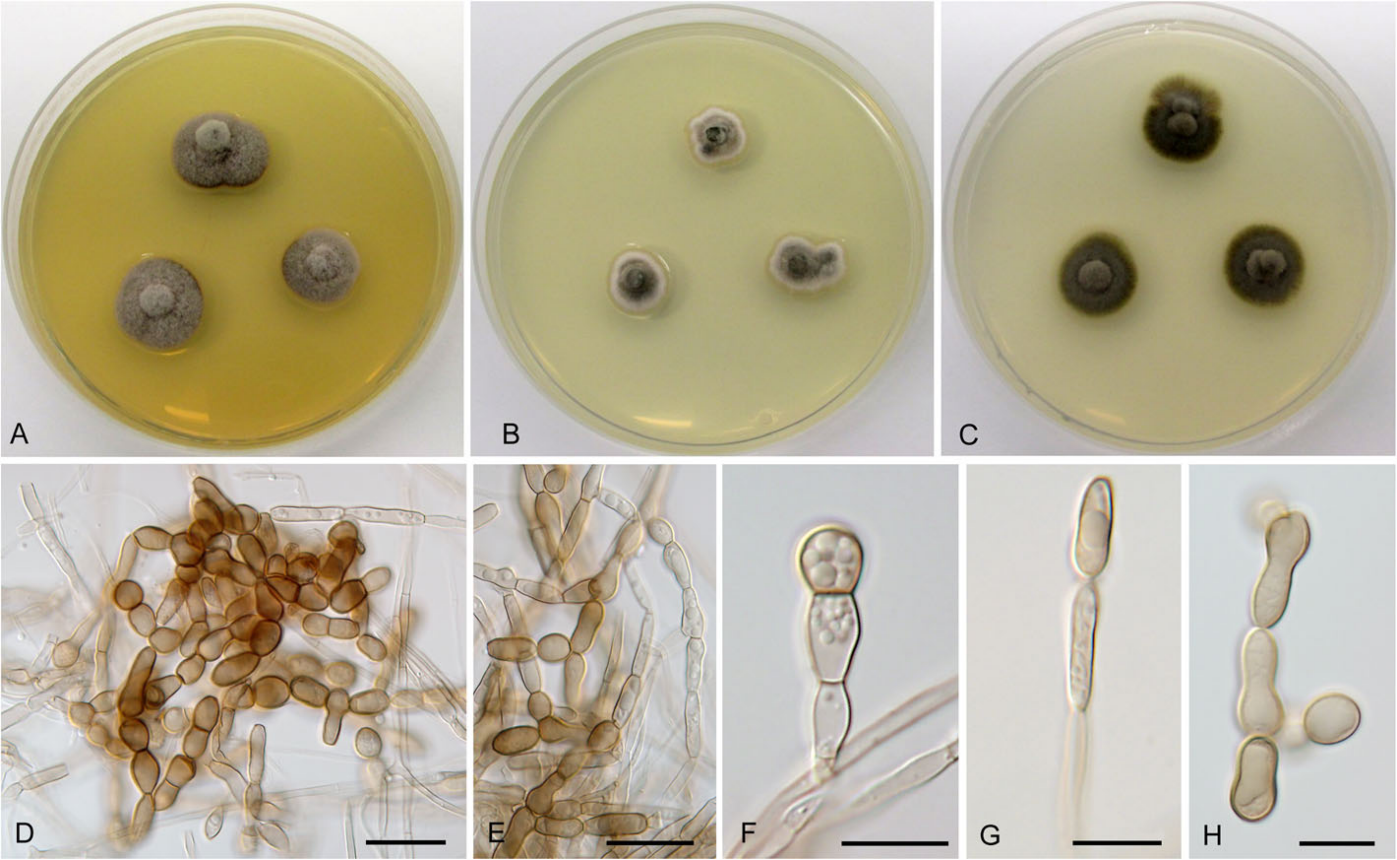
*Cadophora gamsii* (CBS 146379 – ex-type culture). **a** – **c**, 15-day-old colonies on MEA, PDA, and CMA, respectively. **d**, chains of predominantly thick walled conidia. **e**, chains of both thick and thin walled conidia. **f – g**, terminally produced conidia. **h**, detached conidia. Bars: D – E = 20 μm, F – H = 10 μm

**Cadophora gamsii** Koukol & Maciá-Vicente, **sp. nov.** – Figs. 1, 9.

MycoBank: MB 834825.

*Etymology*: Named in honour of Walter Gams (1934–2017) who contributed significantly to the study of the genera *Cadophora* and *Phialophora*.

*Diagnosis*: Morphologically distinct from *C. echinata* by long and rarely branched chains of variably shaped conidia.

*Type*: **France**: Malbrans, 47.11 N 006.07 E, 543 m a.s.l., endophytic in roots of *M. erraticum*, 4 May 2013, *A.K. Buch & X. Xia* [isol. K. Glynou] (FR 0255184 – holotype; P2437 = CBS 146379 – ex-type cultures; GenBank accessions: ITS = KT269668, *tef1-α =* MN325899, *rpb2* = MN367272).

*Description*: *In culture* – *Colonies* on MEA reaching 12–16 mm diam after 10 d, on PDA and CMA reaching 11 mm and 12–13 mm diam, respectively. *Mycelium* septate, hyphae hyaline to pale brown, hyphae 1.5–2.5 μm wide. *Conidiophores* not developed. *Conidiogenous cells* integrated, terminal, holoblastic, monoblastic, smooth, cylindrical, 2.5–5 μm wide. *Conidia* pale brown rather thin walled to dark brown, thick walled, smooth, mostly non-septate (rarely 1-septate), rather variable in shape, from globose to pyriform, spathulate, fusiform, with distinct constriction in the middle when elongated, forming long chains that are rarely branched; thin walled elongated conidia (10–)14–20(− 22.5) × 3.5–5.5 μm wide (when 1-septate 20 μm long), mean conidium length/width ratio 3.9:1; thick walled conidia 7–14.5(− 18.5) × 5–9 μm wide, mean conidium length/width ratio 1.7:1.

*Additional material studied*: **France**: Malbrans, 47.11 N 006.07 E, 543 m a.s.l., endophytic in roots of *M. erraticum*, 4 May 2013, *A.K. Buch & X. Xia* [isol. K. Glynou] (P2440 = CBS 146379 – cultures).

*Note*: *Cadophora gamsii* produces long chains of conidia of variable shapes similarly to *C. echinata*, where these chains branch and may form large sclerotia-like bodies. However, these structures may be also interpreted as inflated hyphal segments (chlamydospores). In *C. orchidicola* they are formed together with regular conidia. These structures presumably do not disperse very far, but rather survive close by and so may have a dormancy function similar to the microsclerotia of dothidealean fungi (Tsuneda et al. 2001).

**Fig. 10:**
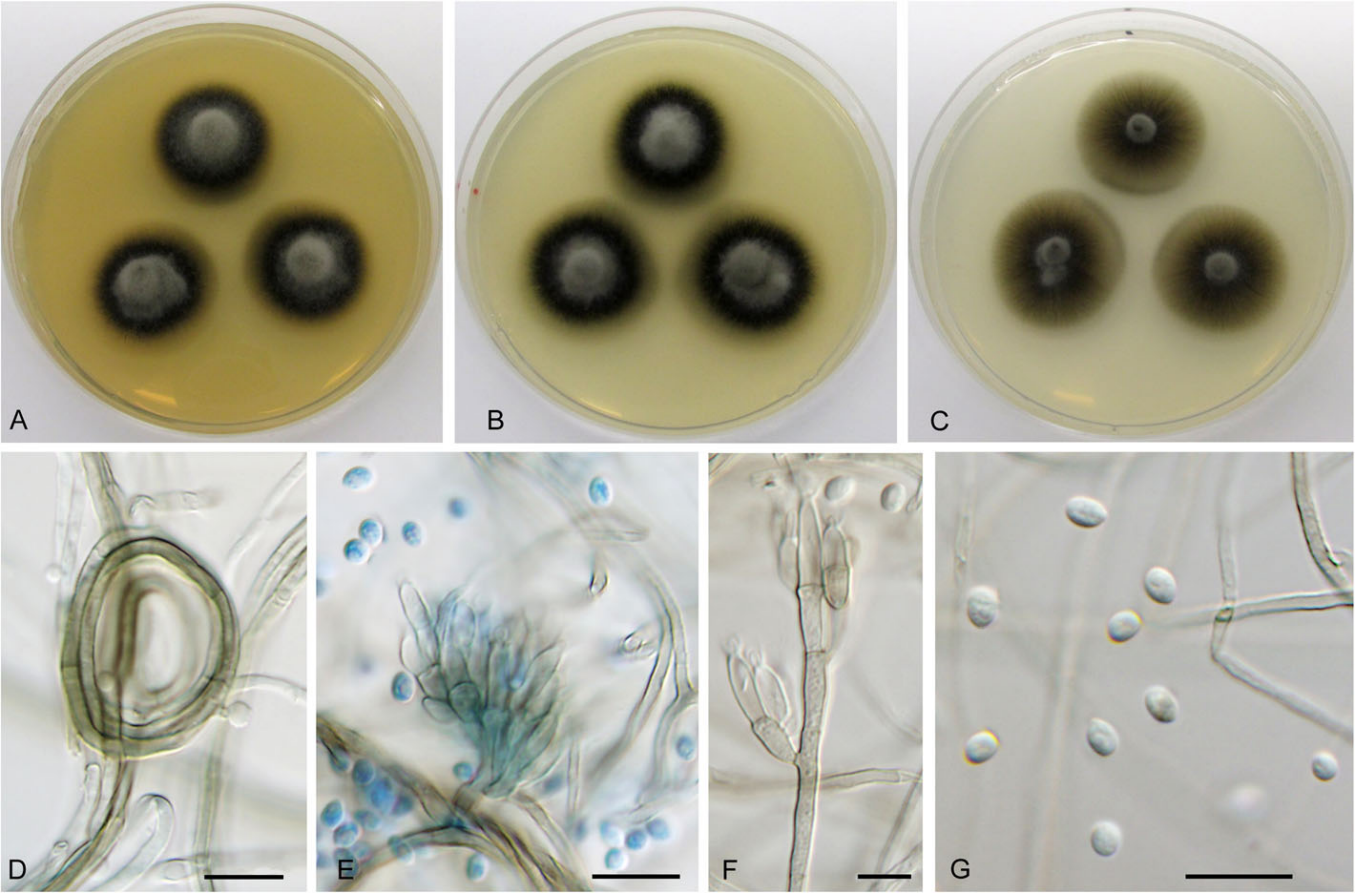
*Cadophora* cf. *interclivum* (isolate P2973). **a** – **c** 15-day-old colonies on MEA, PDA, and CMA, respectively. **d** hyphal coil. **e** fascicle of phialides with conidia (mounted in lactic acid with cotton blue). **f**, terminal and lateral fascicles of phialides. **g**, conidia. Bars: 10 μm

**Cadophora** cf. **interclivum** – Figs. 1, 10*.*

*Description*: *In culture* – *Colonies* on MEA reaching 16–17 mm diam after 10 d, on PDA and CMA reaching 17–18 mm and 17–18 mm diam, respectively. *Mycelium* septate, hyphae hyaline to dark brown, frequently forming coils, hyphae 2–2.5 μm wide. *Conidiophores* straight, bearing single or multiple phialides arranged in intercalary or terminal fascicles. *Conidiogenous cells* phialidic, slightly darker, 6–10 μm long and 2–3 μm wide in the upper part terminated by a collarette 2–3.5 μm long. *Conidia* ellipsoid to ovate with slightly prominent basis, hyaline, with smooth wall, 3–4.5 × 2–3 μm wide, forming clusters but not slimy heads, mean conidium length/width ratio 1.4:1.

*Specimens examined*: **Germany**: Darstadt, 49.68 N 010.00 E, 278 m a.s.l., endophytic in roots of *M. erraticum*, 6 June 2013, *K. Glynou & J.G. Maciá-Vicente* [isol. K. Glynou] (FR 0255220; P2973 – culture). – **Spain**: Puebla de Don Fadrique, 38.04 N 002.48 W, 1630 m a.s.l., endophytic in roots of *M. perfoliatum*, 2 May 2013, *J.G. Maciá-Vicente* [isol. K. Glynou] (FR 0255149; P1396 – culture). – **Germany**: Darstadt, 49.68 N 010.00 E, 278 m a.s.l., endophytic in roots of *M. erraticum*, 6 June 2013, *K. Glynou & J.G. Maciá-Vicente* [isol. K. Glynou] (P2816 – culture).

*Note*: In total, 25 strains clustered with three sequences of *C. interclivum* (including the ex-type strain). Both phenotypic and molecular characteristics were difficult to interpret in concordance with the description of *C. interclivum* by Walsh et al. (2018). Our strains produced slightly darker *Phialocephala*-like phialides with distinct wide open collarettes at the tip of straight conidiophores (Fig. 9), i.e. different from cylindrical conidiogenous cells with cylindrical collarettes and conidia aggregated in slimy heads of *C. interclivum* (Walsh et al. 2018). In analyses of individual genetic loci, only sequence data of the intron in the *tef1-α* gene enabled to distinguish two clades referring to *C. interclivum* and *C. meredithiae* with medium support (PP = 0.87, data not shown). In another analysis, the strains were mixed and no clades were clearly delimited. A similar phenomenon was recorded by Walsh et al. (2018), who found two well supported lineages only when analyzing sequences of genes coding the RNA polymerase II largest subunit (*rpb1*) and β-tubulin. Unfortunately, we did not amplify these markers. Obviously, the lineage *C. interclivum*/*C. meredithiae* contains multiple, hitherto unrecognized root colonizing species, and commonly used markers, such as ITS or *rpb2*, have limited ability to distinguish them.

**Fig. 11:**
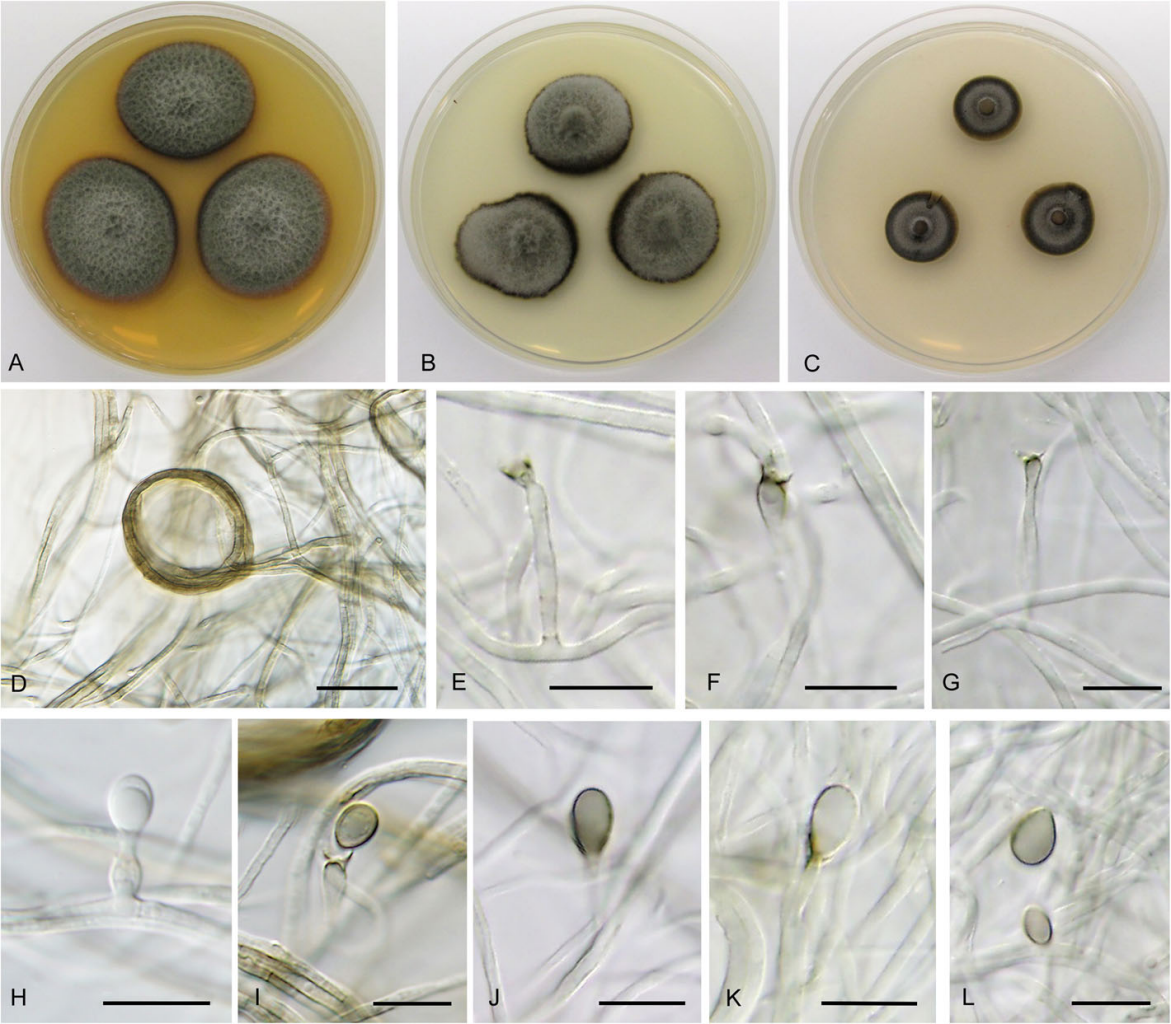
*Cadophora obovata* (CBS 146374 – ex-type culture). **a** – **c** 15-day-old colonies on MEA, PDA, and CMA, respectively. **d** hyphal coil. **e** – **g** conidiogenous cells. **h** young conidiogenous cell producing conidium. **i** conidiogenous cell releasing conidium. **j – l** conidia still attached to conidiogenous cell or free. Bars: D = 20 μm, E – L = 10 μm

**Cadophora obovata** Koukol & Maciá-Vicente, **sp. nov**. – Figs. 1, 11.

MycoBank: MB 834827.

*Etymology*: After the obovate shape of conidia.

*Diagnosis*: Morphologically distinct in having obovate conidia produced from monoblastic phialide-like conidiogenous cells.

*Type*: **Germany**: Ettringen, 50.37 N 007.22 E, 504 m a.s.l., endophytic in roots of *M. erraticum*, 9 May 2013, *M. Thines* [isol. K. Glynou] (FR 0255171 – holotype; P1963 = CBS 146374 – ex-type cultures; GenBank accessions: ITS = KT269230, LSU = MN339384, *tef1-α* = MN325888, *rpb2* = MN367298).

*Description*: *In culture* – *Colonies* on MEA reaching 27–30 mm diam after 10 d, on PDA and CMA reaching 22–25 mm and 15 mm diam, respectively. *Mycelium* septate, hyphae hyaline to dark brown, frequently forming coils or hyphal fascicles 15 μm thick, hyphae 1.5–2.5 μm wide. *Conidiophores* absent. *Conidiogenous cells* holoblastic, monoblastic, obconical, 17–22 μm long and 2–3 μm wide in the upper part terminated by a collarette 2–3.5 μm long. *Conidia* obovate, pale grey-brown, with smooth wall, 6–8(− 9.5) × 3.5–6 μm, mean conidium length/width ratio 1.5:1.

*Additional material examined*: **France**: Saint-Georges-Armont, 47.41 N 006.56 E, 285 m a.s.l., endophytic in roots of *M. erraticum*, 19 April 2013, *J.G. Maciá-Vicente* (FR 0255142; P1101 = CBS 146359 – cultures) **– Germany**: Ettringen, 50.37 N 007.22 E, 504 m a.s.l., endophytic in roots of *M. erraticum*, 9 May 2013, *M. Thines* [isol. K. Glynou] (FR 0255174; P1978 – culture); ibidem (FR 0255176; P1992 – culture).

*Note:* This newly described species is distinct from other *Cadophora* species in having a putatively monoblastic conidiogenous cell. Although the cell resembles a phialide in having a basal swelling of the conidiogenous cell and an irregular collarette-like structure, no subsequent enteroblastic conidiogenesis typical for phialides was observed. Giving its position in the phylogeny, this type of conidiogenous cell seems to represent a retrogression of enteroblastic phialidic conidiogenesis.

**Fig. 12:**
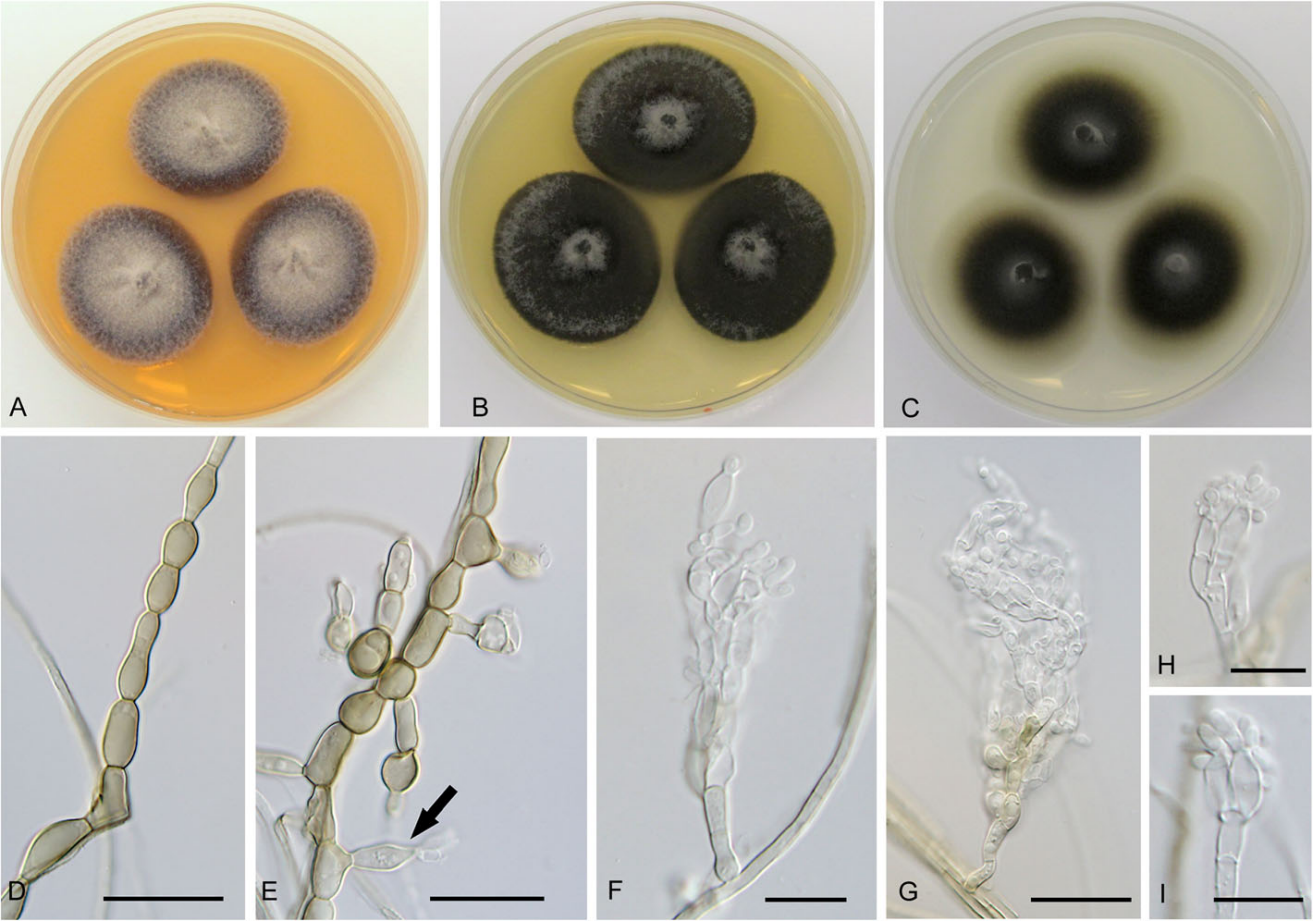
*Cadophora* cf. *orchidicola* (isolate P1854). **a** – **c**, 15-day-old colonies on MEA, PDA, and CMA, respectively. **d – e**, inflated hyphal segments (arrow points to a conidiogenous cell). **f – i**, conidiophores with fascicles of conidiogenous cells. Bars: D – G = 20 μm, H – I = 10 μm

**Cadophora** cf. **orchidicola** – Figs. 1, 12.

*Description*: *In culture* – *Colonies* on MEA reaching 30 mm diam after 10 d, on PDA and CMA reaching 32–33 mm and 29–30 mm diam, respectively. *Mycelium* septate, hyphae subhyaline to dark brown, smooth to verrucose, occasionally forming coils and chains of inflated hyphal segments, rarely branched, 6 μm thick, hyphae 1.5–2.5 μm wide. *Conidiophores* straight or flexuous, hyaline, branched or unbranched, bearing single conidiogenous cells intercalary or multiple arranged in terminal fascicles. *Conidiogenous cells* cylindrical, slightly conical or inflated in lower part, presumably enteroblastic, but without apparent collarette, 6–9.5(− 12) μm long and 2.5–5 μm wide. *Conidia* produced sympodially, ellipsoid, hyaline, with smooth wall, 3–5 × 1.5–2.5 μm wide, forming clusters of 2–4 on the top of the conidiogenous cell, the mean conidium length/width ratio 1.9:1.

*Specimens examined*: **Bulgaria**, Begunovtsi, 42.70 N 022.83 E, 770 m a.s.l., endophytic in roots of *M. erraticum*, 14 May 2013, *T. Ali & S. Ploch* [isol. K. Glynou] (FR 0255161; P1854 = CBS 146372 – cultures).

*Note:* The identification of this clade formed by 23 isolates sequenced in this study is only tentative due to ambiguous evidence from phenotypic and molecular data. A representative strain of this clade intensively sporulating in culture (P1854) was markedly similar to the description of *C. orchidicola*. This species originally described from roots of the orchid *Calypso bulbosa* was characterized as producing “slightly swollen conidiogenous cells” and “terminal conidia produced sympodially” without apparent scars (Currah et al. 1987). Our observations confirmed phialide-like conidiogenous cells, but without distinct collarette; i.e. we were not able to identify the exact mode of conidiogenesis. Also the dimensions of conidia were almost identical to those provided by Currah et al. (1987), i.e., 3–5(− 7) × 1–3 μm. However, reliable identification based on sequence data was not possible. Although the ex-type strain UAMH 5422 is available, *C. orchidicola* is not represented by any ex-type sequence in GenBank. Instead, the database contains more than 380 sequences of *Cadophora*/*Leptodontidium orchidicola*. BLAST searches performed with representative sequences from this clade indicated multiple matches with similarities of 94.5–99.5%, indicating substantial inconsistency in species delimitation.

**Fig. 13:**
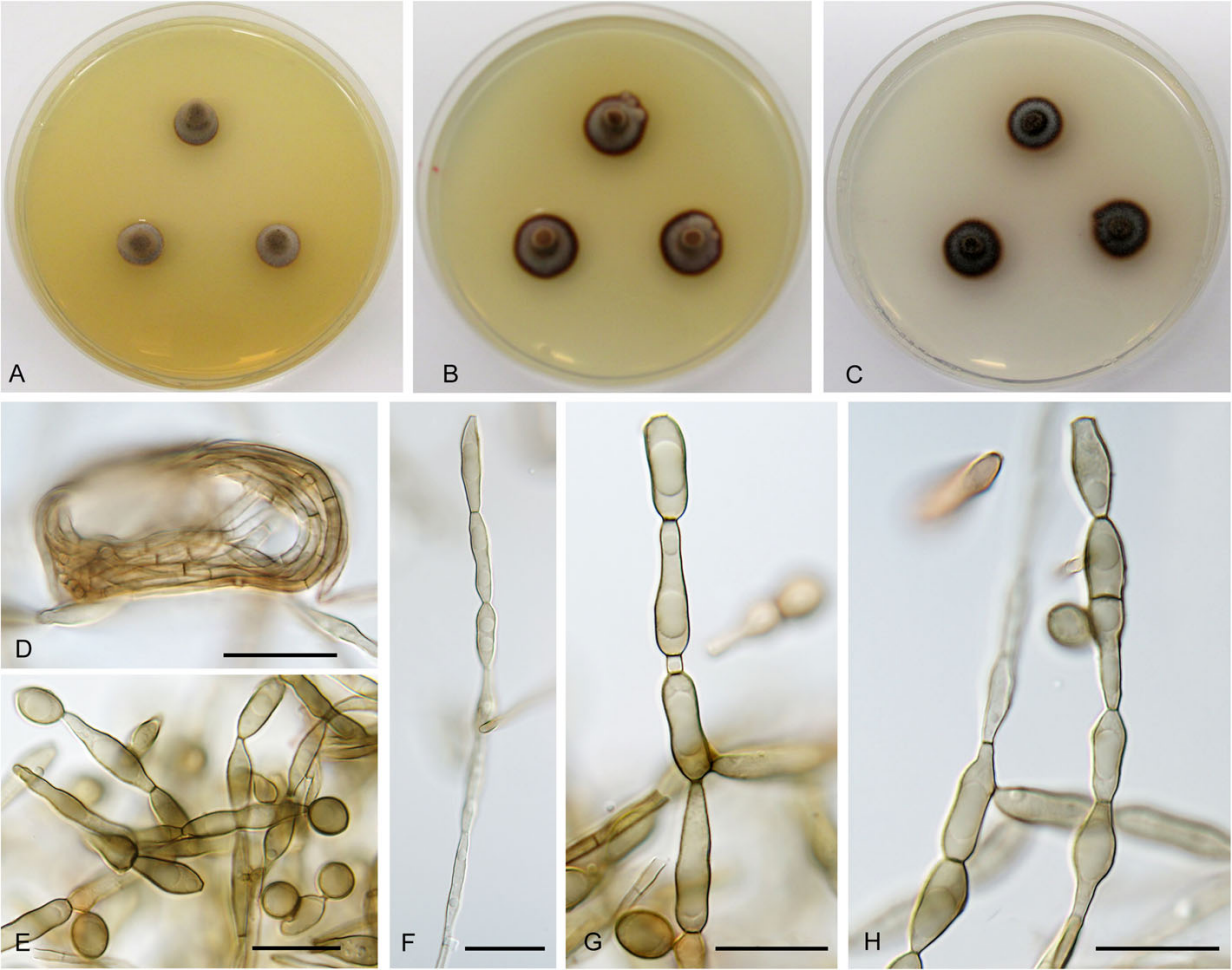
*Cadophora variabilis* (CBS 146360 – ex-type culture). **a** – **c**, 15-day-old colonies on MEA, PDA, and CMA, respectively. **d**, hyphal coil. **e** – **h**, conidial chains. Bars: 20 μm

**Cadophora variabilis** Koukol & Maciá-Vicente, **sp. nov**. – Figs. 1, 13.

MycoBank: MB 834829.

*Etymology*: After the production of conidia of variable shapes.

*Diagnosis*: Morphologically very similar to *C. antarctica*, but with ramoconidia up to 25.5 μm and conidia up to 27 μm long.

*Type*: **Croatia**: Jasenice, 44.24 N, 015.54 E, 760 m a.s.l., endophytic in roots of *M. perfoliatum*, 4 May 2013, *T. Ali* [isol. K. Glynou] (FR 0255203 – holotype; P1176 = CBS 146360 – ex-type cultures; GenBank accessions: ITS = KT268493, LSU = MK539845, *tef1-α =* MK550890, *rpb2* = MN367276).

*Description*: *In culture* – *Colonies* on MEA reaching 9–10 mm diam after 10 d, on PDA and CMA reaching 10–11 mm and 9–10 mm diam, respectively. *Mycelium* septate, hyphae hyaline to dark brown, rarely forming coils 55 μm diam, hyphae 2–3 μm wide. *Conidiophores* not developed. *Conidiogenous cells* integrated, terminal, holoblastic, monoblastic, smooth, cylindrical, 3–5 μm wide. *Ramoconidia* holoblastic non-septate, pale brown to hyaline, smooth, straight, fusiform, frequently constricted in the middle, (16–)18–23.5(− 25.5) × 5–7.5 μm. *Conidia* pale brown to hyaline, smooth, mostly non-septate (rarely 1-septate), rather variable in shape, subglobose to pyriform, obovate or fusiform, with a distinct constriction in the middle when elongated, forming chains (rarely with cylindrical connective or aberrant conidium), (8.5–)11–21(− 27) × 5.5–9 μm, mean conidium length/width ratio 2.2:1.

*Additional material examined.***Spain**: Puebla de Don Fadrique, 38.04 N, 002.48 W, 1630 m a.s.l., endophytic in roots of *M. perfoliatum*, 2 May 2013, *J.G. Maciá-Vicente* [isol. K. Glynou] (FR 0255145; P1331 = CBS 146364 – cultures).

*Note: Cadophora variabilis* is very similar to *C. antarctica*, which also produces chains of ramoconidia and conidia on holoblastic conidiogenous cells. However, the ramoconidia and conidia of *C. variabilis* are larger than those of *C. antarctica*. Interestingly, these two species are very distant in the phylogeny suggesting that this similar morphology has resulted from convergence.

## DISCUSSION

The roots of non-mycorrhizal *Brassicaceae* harbour an unexpected diversity of helotialean endophytes, among other fungi from diverse lineages (Glynou et al. 2016, 2018a). Our screening of only a few plant species, mainly from the genus *Microthlaspi*, yielded seven *Cadophora* species new to science and six additional lineages that also are likely novel species, altogether representing more than a half of the species included so far in the genus. Our results clearly show that *Cadophora* as currently circumscribed is paraphyletic, sharing a common ancestor with species recognized in other genera, and evolved into diverse morphologies and ecological preferences while simultaneously converging in many of their phenotypic characters. Even though most of the species described herein appear to be somewhat rare, they often display relatively wide distribution ranges across the northern hemisphere, mainly in Europe. Some species, however, may be strictly endemic with a very limited distribution range.

### Taxonomy

The paraphyly we found in the genus *Cadophora* had been previously reported by Day et al. (2012), who identified *Cadophora* species distributed across three different clades based on an ITS phylogeny: one containing *Cadophora* species together with species in *Phialocephala* and *Mollisia* (clade A in Day et al. 2012), another also containing species of *Chloridium* and *Meliniomyces* (clade B), and a third comprising most *Cadophora* species, including the type of *C. fastigiata* (clade C). With the combination of *C. hiberna* into *Phialocephala* by Day et al. (2012), and of *C. finlandica* into *Hyaloscypha* by Fehrer et al. (2019), the genus apparently became monophyletic. This was supported by recent studies (Linnakoski et al. 2018; Bien and Damm 2020) in which phylogenies were biased by excluding genera closely related to *Cadophora*. The final dataset assembled in our study not only expands several times the clade C of Day et al. (2012), but also provides a clear support for the paraphyly of *Cadophora*. Species of the genera *Collembolispora*, *Helgardia*, *Mycochaetophora*, *Oculimacula*, *Rhexocercosporidium*, and *Rhynchosporium*, are interspersed between the clades formed by *Cadophora* species (Fig. 1). The ITS sequences alone are not enough to differentiate species within this group, and even single coding-gene phylogenies may result in misleading information. Interestingly, the same gene may have different taxonomic value for different *Cadophora* clades. Whilst Walsh et al. (2018) found clear differentiation of *C. interclivum* and *C. meredithiae* based on the β-tubulin gene, Bien and Damm (2020) suspected the same gene of having paralogues in *C. bubakii*, *C. obscura* and *C. viticola*. The phylogenetic placement of *Graphium rubrum*, represented by a sequence from the ex-type strain CBS 210.34, next to *C. malorum,* is most probably misleading as that strain does not seem to truly originate from the type (Harrington et al. 2001; Harrington and McNew 2003).

Based on our multi-gene phylogeny, we consider that *Cadophora s. str*. forms a distinct basal clade comprising the type of the genus, *C. fastigita* (represented by strain CBS 307.48), together with *C. melinii*, *C. margaritata*, *C. novi-eboraci*, *C. orientoamericana*, *C. viticola*, *C. ramosa*, and *C. ferruginea*. Although another *C. fastigiata* strain, DAOM 225754, appears in a distant position with reference to *Cadophora s. str.,* we consider that *C. fastigiata* strain CBS 307.48 better represents the type species. This strain was isolated from blue-stained wood in Sweden, just like the species type (Lagerberg et al. 1927), and Schol-Schwartz (1970) noted no morphological differences between both cultures. Later studies also used this strain as a representative of *C. fastigiata* (Bien and Damm 2020) or even have considered it, erroneously, derived from the type (Travadon et al. 2015; Crous et al. 2017; Linnakoski et al. 2018).

The paraphyly in the genus *Cadophora*, evidenced here, deserves addressing, possibly most efficiently by splitting the genus into a minimum of three genera to accommodate the clades containing: (1) *Cadophora s. str*.; (2) *C. orchidicola* and *C. echinata* (while simultaneously combining and taking the generic name from *Collembolispora*); and (3) *C. interclivum*, *C. meredithiae*, *C. luteo-olivacea*, *C. malorum*, and *C. helianthi*. In this context, *C. antarctica*, *C. lacrimiformis* and *C. fascicularis* should be combined into *Mycochaetophora*. Nevertheless, we refrain from undertaking this drastic restructuring here, owing to the lack of established, morphological generic concepts, i.e., concerning the corresponding type species that belong to the *Cadophora s. lat*. relationship—as well as of sequence data of further sexual species that would need to be considered for such a task. The creation of new generic concepts without a more comprehensive sampling of species could well lead to their future recognition as synonyms, a situation we want to avoid. Further efforts aimed at the discovery of additional new species, above all with sexual morphs, the sequencing of molecular markers from type specimens, and the epitypification, will be necessary for a robust establishment of the phylogenetic relationships among the lineages thus far constituting *Cadophora* and allied genera.

### Relationships between morphological and phylogenetical data

Day et al. (2012) hypothesized that conidiogenesis in *Cadophora* evolved via simplification of more complex ancestral phialide arrangements, including sclerotium-like structures formed by compact multiple phialides that could serve as diaspores produced on wood surfaces. This evolution could have depended on a higher reliance on conidia for dispersal, and led to the near absence of conidia in *C. orchidicola* (Day et al. 2012), which we corroborated for a large number of isolates related to this species. Our data show a distinct evolution of conidiogenesis across clades, with *Cadophora s. str.* species having evolved from an ancestor with phialidic ontogeny of conidia. *Cadophora s. str.* species thereby differ from the ancestor of other *Cadophora* species, whose conidia were produced holoblastically. Phialidic conidiogenesis appears to have evolved multiple times, giving rise to species in the *C. gregata*, *C. luteo-olivacea*, and *C. malorum* clade (node 6 in Fig. 2). A notable case within this clade is *C. obovata*, which seems to have lost the ability to form further conidia enteroblastically after the first holoblastic conidium is formed. On the contrary, *C. orchidicola*, placed among species producing only holoblastic conidia, has conidiogenesis of an unclear type (Harrington and McNew 2003) showing some similarity to phialidic conidiogenesis. Conidiogenesis thus appears as an important character not only for the identification of *Cadophora* species based on morphology, but also for the evolution of the phenotype. However, in our study many isolates failed to sporulate even after incubation for more than one year at 4 °C, which appears to be required for conidiophore formation in some *Cadophora* and related species (Addy et al. 2005). Other microscopical characters such as the characteristics of conidia are rather variable across the *Cadophora* groups, thus offering limited diagnostic potential, as is the case with macroscopical culture traits such as the shape or texture of the mycelium that are strongly pleomorphic in this group. Overall, morphological characteristics are a poor proxy for phylogenetic affinities in *Cadophora*. The limited availability of diagnostic morphological traits in this and other DSE groups has been responsible for the long-standing difficulty in establishing their systematic positions, that varied across distant taxonomic categories (Gams 2000; Jumpponen 2001; Harrington and McNew 2003; Addy et al. 2005).

### Ecological preferences

The variability in morphological traits makes it difficult to infer the ecological reasons behind the evolution of particular characters, with the only exception of the aquatic genus *Collembollispora*, with branched conidia typical of ingoldian fungi (Dix and Webster 1995). However, the phialidic synanamorph of *Collembolispora barbata* provides a morphological connection to members of *Cadophora*. The first *Cadophora* species was described as a wood-colonizing fungus, and today species in the genus are still frequently isolated from this substrate (Lagerberg et al. 1927; Bills 2004; Gramaje et al. 2011, 2015; Bien and Damm 2020). Our results support such a substrate preference for species of *Cadophora s. str.*, but show a widespread ability to colonize living plant hosts in the remaining clades — particularly roots as a result of the focus of this study. This ability has been confirmed experimentally for several isolates included in our study, representing *C.* cf. *interclivum* (P1686, P1866), *C.* cf. *orchidicola* (P1940), and *C. variabilis* (P1176, P1331), all of which have been shown able to colonize roots from *Brassicaceae* and *Poaceae* hosts without eliciting disease symptoms (Kia et al. 2017, 2018, 2019). Such a root-colonizing habit could indicate that the ancestor of *Cadophora s. lat*. evolved towards a symbiotic lifestyle when split from the ancestor of *Cadophora s. str*. However, reaching definite conclusions about the ecological preferences of the different species is not possible based only on a few representative isolates of many species. In addition, the ability to colonize multiple niches is a common phenomenon in fungi (Selosse et al. 2018), so that the diversity of substrates of *Cadophora* species may just reflect ecological plasticity.

### Distribution patterns

Comparisons of ITS sequences with data available in public databases showed that many of the focal species in this study are related to fungi widely distributed across the Northern Hemisphere, although in most cases nearly identical sequences were exclusively found within Europe. This is in line with previous observations on the distribution of other helotialean DSE in the *Phialocephala fortinii* and *Acephala applanata* species complexes (Grünig et al. 2001; Piercey et al. 2004; Queloz et al. 2011), which show a widespread distribution in northern temperate zones. The absence of similar sequences in the Southern Hemisphere could be caused by biases in the geographic representation of fungal ITS sequence data in GenBank, with several Southern Hemisphere countries being underrepresented as compared to countries in the Northern one. This possibility is, indeed, suggested by a higher representation in the Southern Hemisphere of UNITE SH assigned to *Cadophora* sp. 1, *Cadophora* sp. 4, *Cadophora* sp. 5, *C. echinata*, *C.* cf. *interclivum*, *C. ferruginea*, *C. luteo-olivacea*, *C*. cf. *meredithiae*, and *C. variabilis* (Table S1). However, northern countries such as the USA and China, amongst the most represented by fungal ITS sequences in GenBank, did not yield as many BLAST matches as European countries did, thus adding support to the distribution patterns we observed.

Just considering our isolates, all species with more than two isolates had representatives at separate locations over Europe, while often co-existing in the same site with isolates of other *Cadophora* species. This reproduces the observations of Grünig et al. (2002) and Piercey et al. (2004) on *P. fortinii*, who found individuals from separate locations were more closely related to one another than were individuals collected a few metres apart, and also agrees with similar results with several lineages of dominant root endophytes (Glynou et al. 2017). The mechanisms for such long distance dispersal are unknown, for example whether this is achieved via conidial dispersion or via transportation within plant material as microsclerotia or other diaspores (Currah et al. 1993). We did not observe a correlation between the production of conidia in culture and the breadth of the species distribution ranges, e.g. *C. orchidicola* barely produces conidia but has widespread occurrences, and *C. fascicularis* which produces numerous conidia but has a restricted distribution. In contrast to the wide distribution of most species, *C. fascicularis*, *C. ferruginea*, *Cadophora* sp. 3, *Cadophora* sp. 5, and *Mycochaetophora* sp. show very restricted distribution ranges both worldwide and across Europe, with occurrences in only one or a few close locations. Given the scarcity of isolates and identical sequences available for these species, it is difficult to conclude that the restricted distributions of these species are due to true endemism, with species adapted only to very local conditions, or due to rarity that precludes their capture in ecological studies by current sampling methods. This question will only be settled upon by further sampling activities and descriptions of fungal diversity worldwide.

## CONCLUSIONS

Our dataset is probably the most comprehensive considering currently recognized *Cadophora* species. It shows an unexpectedly high diversity of species in roots of *Microthlaspi* spp., which led to the description of seven species new to science in this study and enabled a new and a more complete view of the life history of members of this helotialean genus to be provided. Our results also show current knowledge gaps in the phylogenetic relationships within *Cadophora* and with other, related lineages. Further sampling efforts to discover new species, as well as further descriptions and sequence data from species types, are necessary to resolve the systematics of this fascinating group of fungi.

## Supplementary information

**Additional file 1: Fig. S1.** Clustering of the focal isolates of this study into operational taxonomic units (OTUs) based on pairwise ITS rDNA sequence similarity at 97, 98 and 99%. The trees represent a subset of the phylogeny shown in Fig. 1, including only the isolates used in this study, with tip labels and colors representing the OTU grouping of isolates at each similarity threshold. The OTU clustering was performed as described in Glynou et al. (2016), representing a standard procedure in fungal community ecology studies. **Fig. S2.** Ancestral character state reconstruction in *Cadophora* and allied species for all morphological and ecological characteristics considered in this study. **Fig. S3.** Principal component analysis (PCA) ordination of isolates according to their quantitative morphological characters, including those removed from Fig. 3. **Fig. S4.** Distribution of all quantitative characters considered in this study across the isolates’ phylogeny. Colors next to tree tips indicate the isolates’ species (see color key). **Fig. S5.** Potential worldwide distribution of all the *Cadophora* species target of this study as inferred by BLAST comparisons against the NCBI GenBank’s nucleotide database (nt), and selected high-throughput ITS amplicon sequencing datasets available at the sequence reads archive (SRA; Table S3). Each map shows the BLAST search results for all the isolates within each species. Points represent the geographic locations of BLAST matches, either from the nt or the SRA database (indicated by point shape). Only BLAST matches with percent identity above 97% are shown, with points color indicating percent identity value.

**Additional file 2: Table S1.** Details of the fungal isolates used in this study, including accession numbers, morphological characters, and data on the geographical location, climate, and soil conditions at the site of origin. **Table S2.** Details of the reference strain sequences included in the phylogenetic analysis. **Table S3.** NCBI data sets of reference ITS sequences used for BLAST comparisons. **Table S4.** Phylogenetic conservation among quantitative traits based on Blomberg’s *K*.

## Data Availability

Living cultures of all isolates have been deposited in the IPF fungal culture collection hosted at Goethe University Frankfurt, and a representative subset of the isolates were also deposited in the CBS culture collection (Westerdijk Fungal Biodiversity Institute, Utrecht, The Netherlands). Dried cultures were deposited in the Herbarium Senckenbergianum Frankfurt am Main (FR). The DNA alignments and the phylogenetic trees are available at TreeBASE under accession S25942. All data and code used in this study is available online at 10.6084/m9.figshare.12287816.
