# Supplementary material for: Brassicaceous roots as an unexpected diversity hot-spot of helotialean endophytes
Source: IMA Fungus. 2020 Aug 11;11:16. doi: 10.1186/s43008-020-00036-w (PMC7419212; doi:10.1186/s43008-020-00036-w)
Supplement: Supplementary file 1 — Additional file 1: Fig. S1. Clustering of the focal isolates of this study into operational taxonomic units (OTUs) based on pairwise ITS rDNA sequence similarity at 97, 98 and 99%. The trees represent a subset of the phylogeny shown in Fig. 1, including only the isolates used in this study, with tip labels and colors representing the OTU grouping of isolates at each similarity threshold. The OTU clustering was performed as described in Glynou et al. (2016), representing a standard procedure in fungal community ecology studies. Fig. S2. Ancestral character state reconstruction in Cadophora and allied species for all morphological and ecological characteristics considered in this study. Fig. S3. Principal component analysis (PCA) ordination of isolates according to their quantitative morphological characters, including those removed from Fig. 3. Fig. S4. Distribution of all quantitative characters considered in this study across the isolates’ phylogeny. Colors next to tree tips indicate the isolates’ species (see color key). Fig. S5. Potential worldwide distribution of all the Cadophora species target of this study as inferred by BLAST comparisons against the NCBI GenBank’s nucleotide database (nt), and selected high-throughput ITS amplicon sequencing datasets available at the sequence reads archive (SRA; Table S3). Each map shows the BLAST search results for all the isolates within each species. Points represent the geographic locations of BLAST matches, either from the nt or the SRA database (indicated by point shape). Only BLAST matches with percent identity above 97% are shown, with points color indicating percent identity value. [file 43008_2020_36_MOESM1_ESM.pdf]

Brassicaceous roots as an unexpected diversity hot-spot of helotialean endophytes

Jose G. Macia-Vicente[1,2], Meike Piepenbring[1,2], Ondřej Koukol[1]

[1]Institute of Ecology, Evolution and Diversity, Goethe University Frankfurt, Max-von-Laue-Str. 13, 60438, Frankfurt am Main, Germany
[2]Integrative Fungal Research Cluster (IPF), Frankfurt am Main, Germany
[3]Department of Botany, Faculty of Science, Charles University, Benátská 2, 12801 Praha 2, Czech Republic

List of Figures

S1 Clustering of the isolates into operational taxonomic units (OTUs) based on pairwise ITS rDNA sequence similarity at 97, 98 and 99 %. 2
S2 Ancestral character state reconstruction in *Cadophora* and allied species. 3
S3 Principal component analysis (PCA) ordination of isolates according to their quantitative morphological characters. 4
S4 Distribution of all quantitative characters considered in this study across the isolates' phylogeny. 5
S5 Potential worldwide distribution of all the *Cadophora* species. 6

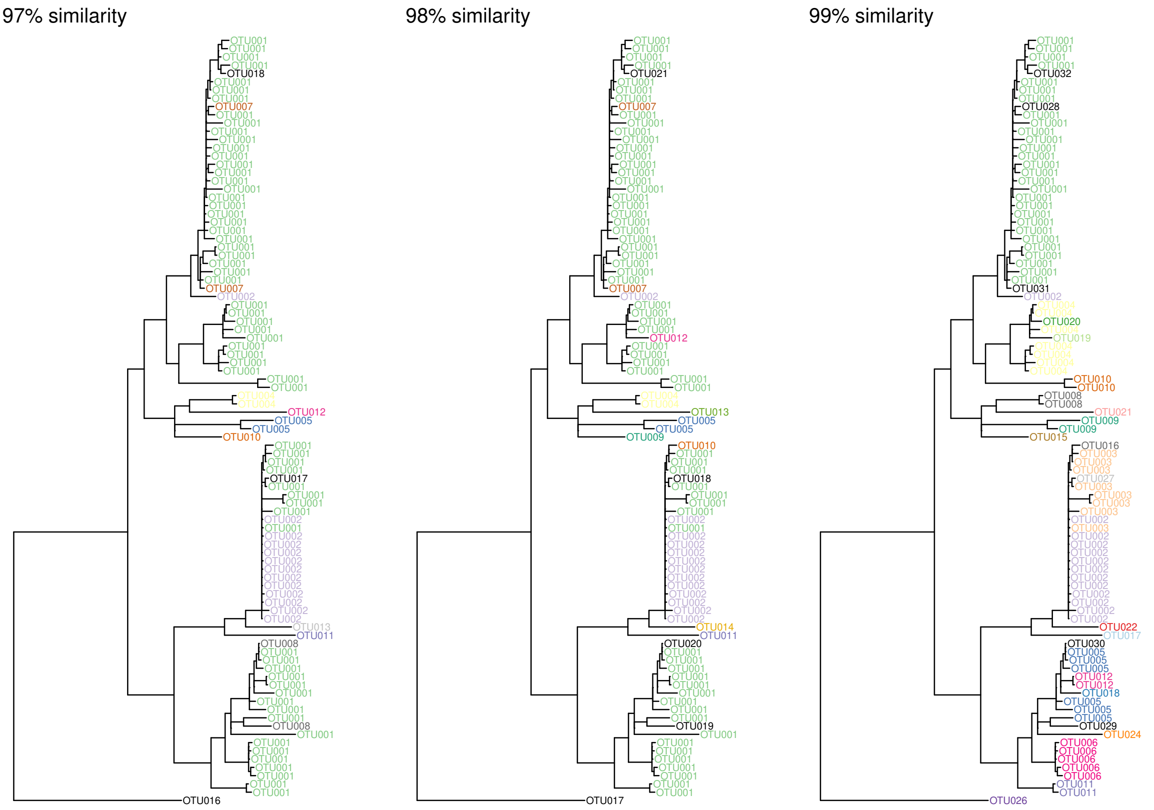

Figure S1: Clustering of the focal isolates of this study into operational taxonomic units (OTUs) based on pairwise ITS rDNA sequence similarity at 97, 98 and 99 %. The trees represent a subset of the phylogeny shown in Fig. 1, including only the isolates used in this study, with tip labels and colors representing the OTU grouping of isolates at each similarity threshold. The OTU clustering was performed as described in Glynou et al. (2016) Environ Microbiol 18: 2418–2434, representing a standard procedure in fungal community ecology studies.

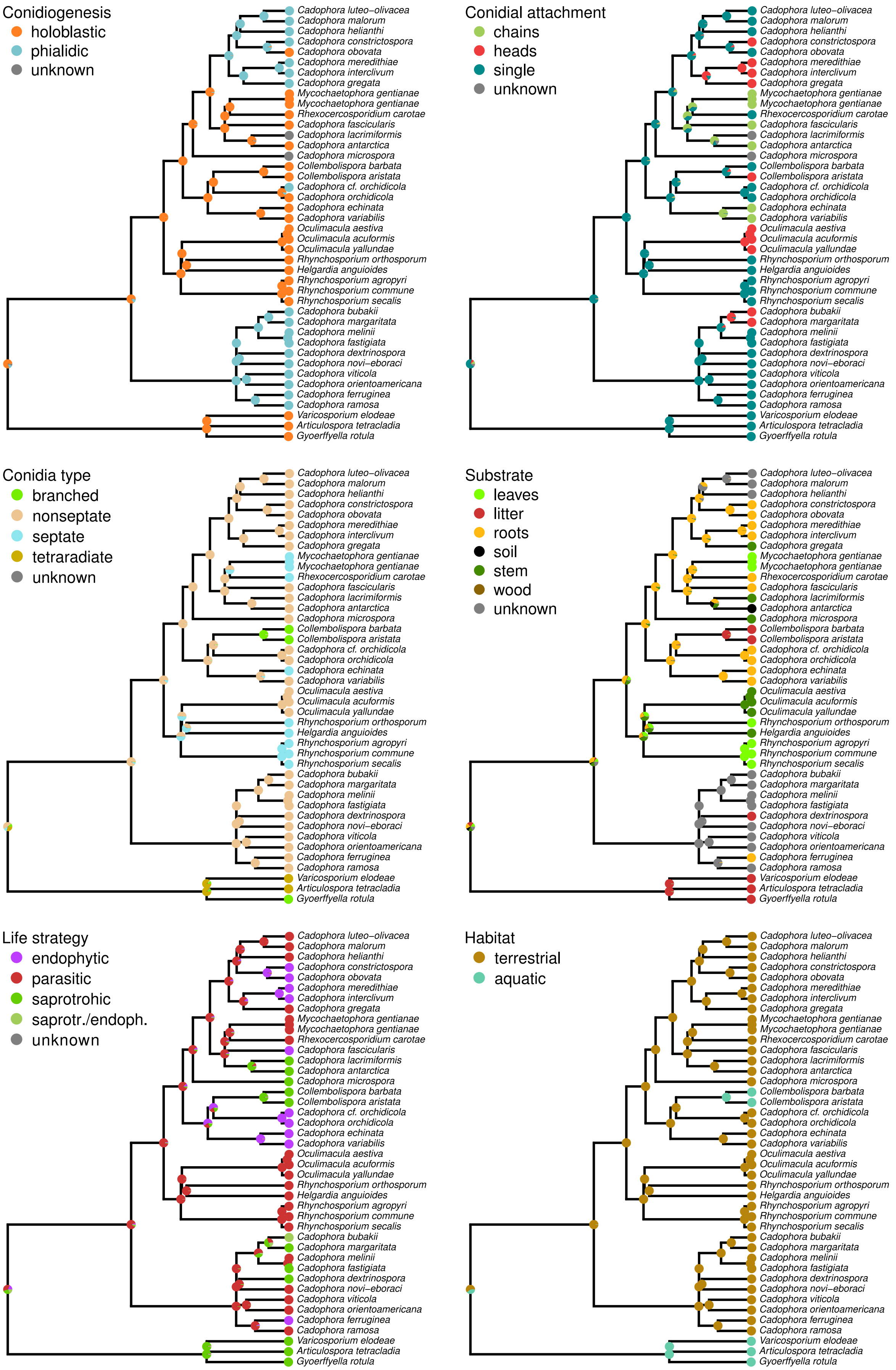

Figure S2: Ancestral character state reconstruction in *Cadophora* and allied species for all morphological and ecological characteristics considered in this study.

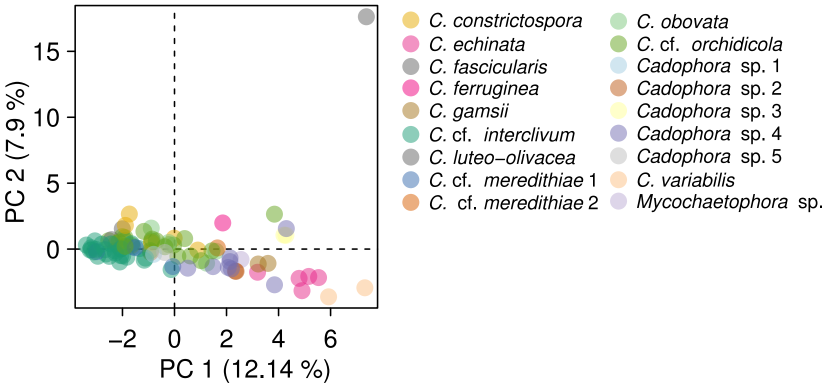

Figure S3: Principal component analysis (PCA) ordination of isolates according to their quantitative morphological characters, including those removed from Fig. 3.

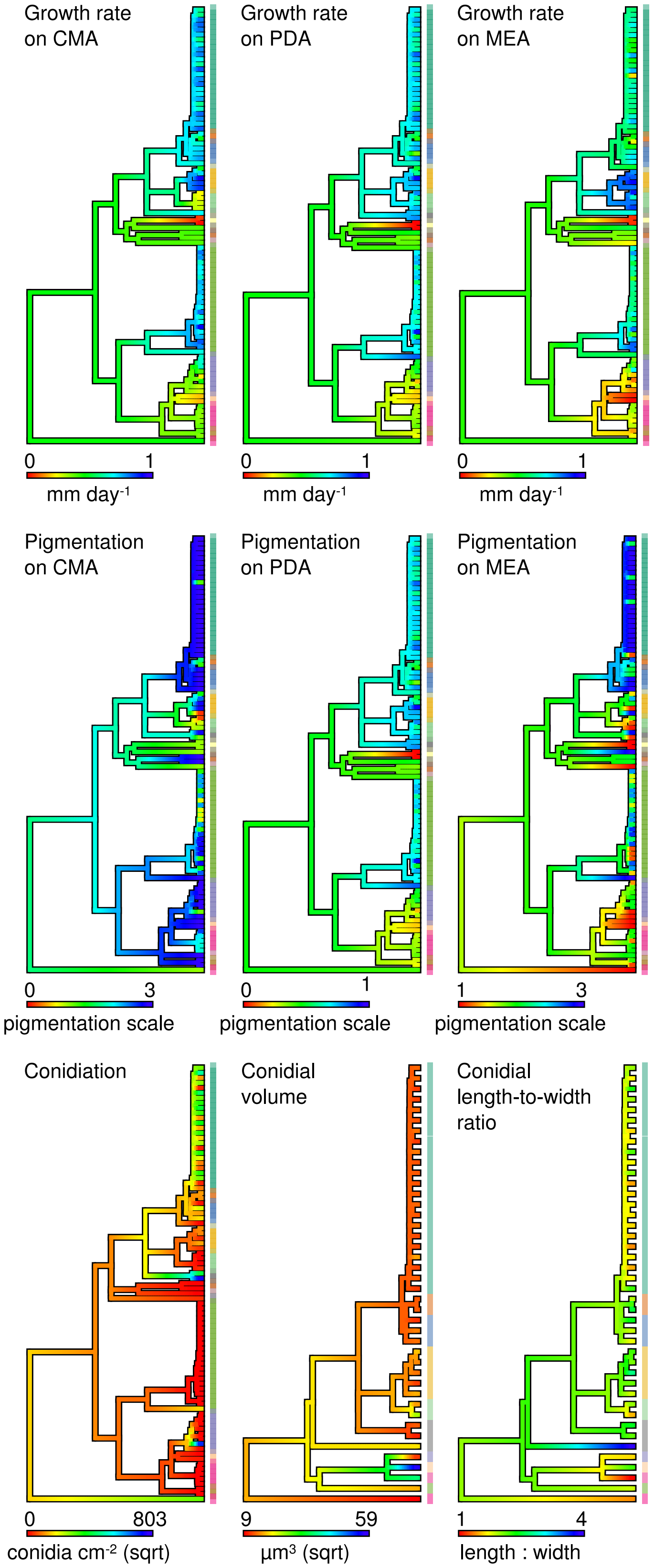

Figure S4: Distribution of all quantitative characters considered in this study across the isolates' phylogeny. Colors next to tree tips indicate the isolates' species (see color key).

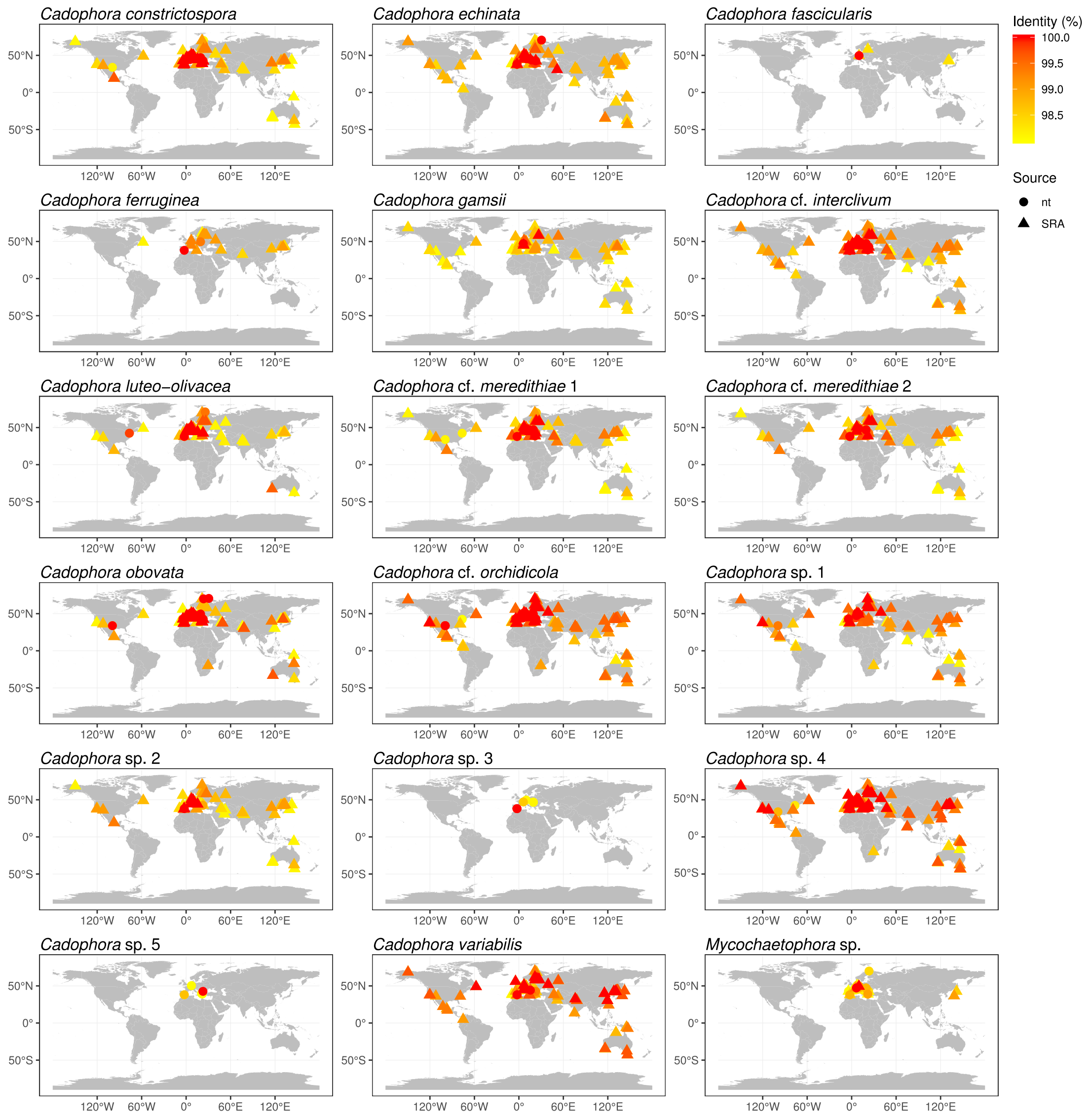

Figure S5: Potential worldwide distribution of all the *Cadophora* species target of this study as inferred by BLAST comparisons against the NCBI GenBank's nucleotide database (nt), and selected high-throughput ITS amplicon sequencing datasets available at the sequence reads archive (SRA; Table S3). Each map shows the BLAST search results for all the isolates within each species. Points represent the geographic locations of BLAST matches, either from the nt or the SRA database (indicated by point shape). Only BLAST matches with percent identity above 97 % are shown, with points color indicating percent identity value.
